# A Unified Approach to Shape and Topological Sensitivity Analysis of Discretized Optimal Design Problems

**DOI:** 10.1007/s00245-023-10016-2

**Published:** 2023-06-09

**Authors:** P. Gangl, M. H. Gfrerer

**Affiliations:** 1 Johann Radon Institute for Computational and Applied Mathematics, Altenberger Straße 69, 4040 Linz, Austria; 2 Institute of Applied Mathematics, Graz University of Technology, Steyrergasse 30/III, 8010 Graz, Austria; 3 Chair of Applied Mathematics (Continuous Optimization), Friedrich Alexander University Erlangen-Nürnberg, Cauerstraße 11, 91058 Erlangen, Germany; 4 Institute of Applied Mechanics, Graz University of Technology, Technikerstrasse 4, 8010 Graz, Austria

**Keywords:** Shape derivative, Topological derivative, Unified sensitivity analysis, Design optimization, Finite element method, MSC 49Q10, MSC 65K10

## Abstract

We introduce a unified sensitivity concept for shape and topological perturbations and perform the sensitivity analysis for a discretized PDE-constrained design optimization problem in two space dimensions. We assume that the design is represented by a piecewise linear and globally continuous level set function on a fixed finite element mesh and relate perturbations of the level set function to perturbations of the shape or topology of the corresponding design. We illustrate the sensitivity analysis for a problem that is constrained by a reaction–diffusion equation and draw connections between our discrete sensitivities and the well-established continuous concepts of shape and topological derivatives. Finally, we verify our sensitivities and illustrate their application in a level-set-based design optimization algorithm where no distinction between shape and topological updates has to be made.

## Introduction

Numerical methods for the design optimization of technical systems are of great interest in science and engineering. Applications include the optimization of mechanical structures [2, 29], electromagnetic devices [5, 19], fluid flow [21], heat dissipation [20] and many more. There exist several different approaches to computational design optimization. On the one hand, shape optimization techniques based on the mathematical concept of shape derivatives [15] can modify boundaries and material interfaces in a smooth way, but typically cannot alter the topology of a design. An exception being the level set method for shape optimization [2] where the design is represented by the zero level set of a design function whose evolution is guided by shape sensitivities via a transport equation. While this approach allows for merging of components, it lacks a nucleation mechanism and is often coupled with the topological derivative concept [26, 30], see e.g. [1, 10]. For a computationally-oriented introduction to these classical level-set based methods, we refer the interested reader to [12]. In the class of density-based topology optimization methods [7], a design is represented by a density function $\rho (x)$ that is allowed to attain any value in the interval [0, 1]. Then, regions with $\rho (x) = 0$ and $\rho (x) = 1$ are interpreted as occupied by material 1 and 2, respectively, while intermediate density values $0<\rho (x) < 1$ are penalized in order to obtain designs that are almost “black-and-white”. One advantage of density based methods is that the system response depends continuously on $\rho$ and the standard notions of derivatives in vector spaces can be applied. Here, interfaces are typically not crisp and there is no measure of optimality with respect to shape variations at the interface. Finally we mention the level-set algorithm for topology optimization introduced in [4], where the design is guided solely by the topological derivative, which however is not defined on the material interfaces. As a consequence, the final designs cannot be shown to be optimal with respect to shape variations at the interface. This aspect has been thoroughly analyzed in [6] where the authors draw a connection to density-based methods and, for two particular problem classes, propose an interpolation scheme which relates the derivative with respect to the density function, to topological and shape derivatives in the interior and on the interface, respectively.

The goal of this paper is to unify the concepts of topological and shape perturbations and to treat design optimization problems by a unified sensitivity, called the *topological-shape derivative*. In this way, we aim at combining topological sensitivity information (related to the topological derivative) in the interior of each subdomain and shape sensitivity information (related to the shape derivative) at the material interface. While the topological derivative is defined as the sensitivity of a design-dependent cost function with respect to the introduction of a small hole or inclusion of different material, the shape derivative is defined as the cost function’s sensitivity with respect to a transformation of the domain. In order to unify these two concepts, we consider a domain description by means of a continuous level set function which attains positive values in one of two subdomains and negative values in the other. Then a perturbation of the level set function in the interior of a subdomain can be related to a topological perturbation, and a perturbation close to the material interface can be seen as a perturbation of the shape of the domain. We remark that this point of view is in alignment with the concept of dilations of points and curves as introduced in [13], see also [14]. In principle, this idea was already followed in [9] and also in the recent work [8] where the discrete shape sensitivity analysis for perturbations of boundaries represented by level set functions is carried out and compared to the classical approach where domains are perturbed by transformations rather than dilations. In these works, however, only the case of shape optimization and no combination with topology optimization is considered. In [22] the author represents domains by level set functions and relates shape and topological derivatives of shape functionals to derivatives with respect to the level set function in a continuous setting without PDE constraints. In contrast to this, we consider PDE-constrained problems, but our analysis is performed on the discrete level, i.e. we follow the paradigm “discretize-then-optimize” for our sensitivity analysis with respect to a level set function.

The rest of this paper is organized as follows: In Sect. 2, we introduce the model problem and the classical concepts of topological and shape derivative in the continuous setting. After presenting the discretized setting in Sect. 3, we proceed to compute the numerical topological-shape derivative of our discretized model problem in Sect. 4. In Sect. 5 we compare the computed sensitivities with the sensitivities obtained by discretizing the continuous formulas. Finally we verify our computed formulas and present optimization results in Sect. 6 before giving a conclusion in Sect. 7.

## Model Problem and Continuous Setting

Let *D* be a given, fixed, open and bounded hold-all domain and $\varOmega \subset D$ an open and measurable subset. Let the boundary of *D* be decomposed into $\Gamma _D, \Gamma _N \subset D$ with ${\overline{\Gamma }}_D \cup {\overline{\Gamma }}_N = \partial D$ and $\Gamma _D \cap \Gamma _N = \emptyset$. In the present paper, we consider a topology optimisation problem with a tracking type cost function

1 $$\begin{aligned} \begin{aligned} g(\varOmega ,u) = c_1 |\varOmega | + c_2 \int _D {\tilde{\alpha }}_\varOmega |u-{\hat{u}}|^2 \;\textrm{d} x \end{aligned} \end{aligned}$$

where ${\hat{u}}\in H^1(D)$ is a given desired state, and $c_1$, $c_2 \in {\mathbb {R}}$ are given constants. The continuous topology optimization problem reads

2a $$\begin{aligned} \min _{\varOmega \in {\mathcal {A}}} \; g(\varOmega ,u),&\end{aligned}$$

2b $$\begin{aligned} \text { subject to}&\nonumber \\ - \lambda _\varOmega \Delta u + \alpha _\varOmega u&= f_\varOmega{} & {} \text {in } D, \end{aligned}$$

2c $$\begin{aligned} u&= g_D \quad{} & {} \text {on } \Gamma _D, \end{aligned}$$

2d $$\begin{aligned} \lambda _\varOmega \partial _n u&= g_N{} & {} \text {on } \Gamma _N, \end{aligned}$$

where

$$\begin{aligned} {\tilde{\alpha }}_\varOmega (x) =&\chi _\varOmega (x){\tilde{\alpha }}_1 + \chi _{D \setminus \varOmega }(x) {\tilde{\alpha }}_2, \qquad \lambda _\varOmega (x) = \chi _\varOmega (x)\lambda _1 + \chi _{D \setminus \varOmega }(x) \lambda _2,\\ \alpha _\varOmega (x) =&\chi _\varOmega (x)\alpha _1 + \chi _{D \setminus \varOmega }(x) \alpha _2, \qquad f_\varOmega (x) = \chi _\varOmega (x)f_1 + \chi _{D \setminus \varOmega }(x) f_2, \end{aligned}$$

for some constants $\lambda _1, \lambda _2 > 0$, $\alpha _1, \alpha _2, {\tilde{\alpha }}_1, {\tilde{\alpha }}_2 \ge 0$ and $f_1, f_2 \in \mathbb R$ with $\chi _S$ the characteristic function of a set *S*,

$$\begin{aligned} \chi _S(x) = {\left\{ \begin{array}{ll} 1, &{} x \in S, \\ 0, &{} \text{ else }. \end{array}\right. } \end{aligned}$$

Here, ${\mathcal {A}}$ denotes a set of admissible subsets of *D*, and the data $g_D \in H^{1/2}(\Gamma _D)$, $g_N \in L^2(\Gamma _N)$ are given. The weak formulation of the PDE constraint reads

3 $$\begin{aligned} \text{ Find } u\in V_g:=\{v \in H^1(D): v = g_D \text{ on } \Gamma _D \} \text{ such } \text{ that } \nonumber \\ \int _D \lambda _\varOmega \nabla u \cdot \nabla v + \alpha _\varOmega u v \; \text{ d }x = \int _D f_\varOmega v \; \text{ d }x \quad \text{ for } \text{ all } v \in V_0 \end{aligned}$$

with $V_0 = \{v \in H^1(D): v = 0 \text{ on } \Gamma _D \}$. We assume that either $|\Gamma _D|>0$ or $\alpha _1, \alpha _2>0$ such that, for given $\varOmega \in {\mathcal {A}}$, (3) admits a unique solution which we denote by $u(\varOmega )$. We introduce the reduced cost function ${\mathfrak {g}}(\varOmega ):= g(\varOmega , u(\varOmega ))$.

### Classical Topological Derivative

Let $\omega \subset {\mathbb {R}}^d$ with $0 \in \omega$. For a point $z \in \varOmega \cup (D {\setminus } {\overline{\varOmega }})$, let $\omega _\varepsilon := z + \varepsilon \omega$ denote a perturbation of the domain around *z* of (small enough) size $\varepsilon$ and of shape $\omega$. The continuous topological derivative of the shape function ${\mathfrak {g}}={\mathfrak {g}}(\varOmega )$ is defined by

4 $$\begin{aligned} d_T{\mathfrak {g}}(\varOmega )(z) = {\left\{ \begin{array}{ll} \lim _{\varepsilon \searrow 0}\frac{{\mathfrak {g}}(\varOmega \cup \omega _\varepsilon )-{\mathfrak {g}}(\varOmega )}{|\omega _\varepsilon |} \quad \text {for } z \in D \setminus {\overline{\varOmega }}, \\ \lim _{\varepsilon \searrow 0}\frac{{\mathfrak {g}}(\varOmega \setminus {\bar{\omega }}_\varepsilon )-{\mathfrak {g}}(\varOmega )}{|\omega _\varepsilon |} \quad \text {for } z \in \varOmega . \end{array}\right. } \end{aligned}$$

Note that the topological derivative is not defined for points $z \in \partial \varOmega$ on the material interface. For problem (2) we obtain for $z \in D {\setminus } {\overline{\varOmega }}$

5 $$\begin{aligned} \begin{aligned} d_T{\mathfrak {g}}(\varOmega )(z) =&c_1+c_2({\tilde{\alpha }}_1 - {\tilde{\alpha }}_2)(u(z)-{\hat{u}}(z))^2 \\&+ 2\lambda _2\frac{\lambda _1-\lambda _2}{\lambda _1+\lambda _2}\nabla u(z) \cdot \nabla p(z) +(\alpha _1-\alpha _2)u(z)p(z)- ( f_1 - f_2)p(z), \end{aligned} \end{aligned}$$

whereas for $$z \in \varOmega $$

6 $$\begin{aligned} \begin{aligned} d_T{\mathfrak {g}}(\varOmega )(z) =&-c_1+c_2 ({\tilde{\alpha }}_2 - {\tilde{\alpha }}_1)(u(z)-{\hat{u}}(z))^2 \\&+ 2\lambda _1\frac{\lambda _2-\lambda _1}{\lambda _1+\lambda _2}\nabla u(z) \cdot \nabla p(z) +(\alpha _2-\alpha _1)u(z)p(z)- ( f_2 - f_1)p(z), \end{aligned} \end{aligned}$$

see, e.g. [3].

### Classical Shape Derivative

We recall the definition of the classical shape derivative as well as its formula for our model problem (1)–(2). Given an admissible shape $\varOmega \in {\mathcal {A}}$ and a smooth vector field $V \in C_c^\infty (D)$ that is compactly supported in *D*, we define the perturbed domain

7 $$\begin{aligned} \varOmega _t = (\text {id}+tV)(\varOmega ), \end{aligned}$$

for a small perturbation parameter $t > 0$ where $\text {id}:\mathbb R^d \rightarrow {\mathbb {R}}^d$ denotes the identity operator. The classical shape derivative of ${\mathfrak {g}}$ at $\varOmega$ with respect to *V* is then given by

8 $$\begin{aligned} d_S{\mathfrak {g}}(\varOmega )(V) = \lim _{t\searrow 0} \frac{{\mathfrak {g}}(\varOmega _t)-{\mathfrak {g}}(\varOmega )}{t} \end{aligned}$$

if this limit exists and the mapping $V \mapsto d_S{\mathfrak {g}}(\varOmega )(V)$ is linear and continuous. Under suitable assumptions it can be shown that this shape derivative admits the tensor representation

9 $$\begin{aligned} d_S{\mathfrak {g}}(\varOmega )(V) =\int _D {\mathcal {S}}_1^\varOmega : \partial V + {\mathcal {S}}_0^\varOmega \cdot V dx, \end{aligned}$$

for some tensors ${\mathcal {S}}_0^\varOmega \in L^1(D, {\mathbb {R}}^{d} )$, ${\mathcal {S}}_1^\varOmega \in L^1(D, {\mathbb {R}}^{d\times d} )$ [23]. Here, $\partial V$ denotes the Jacobian of the vector field *V*. The structure theorem of Hadamard-Zolésio [15, pp. 480–481] states that under certain smoothness assumptions the shape derivative of a shape function ${\mathfrak {g}}$ with respect to a vector field *V* can always be written as an integral over the boundary of a scalar function *L* times the normal component of *V*, i.e.,

10 $$\begin{aligned} d_S{\mathfrak {g}}(\varOmega )(V) = \int _{\partial \varOmega } L \,(V \cdot n) \; \text{ d }s \end{aligned}$$

where *n* denotes the unit normal vector pointing out of $\varOmega$. For problem (2) one obtains [23]

11 $$\begin{aligned} {\mathcal {S}}_1^\varOmega&= (c_1 \chi _\varOmega + c_2 {\tilde{\alpha }}_\varOmega (u-{\hat{u}})^2 + \lambda _\varOmega \nabla u \cdot \nabla p + \alpha _\varOmega u p - f_\varOmega p) I \nonumber \\&\quad - \lambda _\varOmega \nabla u \otimes \nabla p - \lambda _\varOmega \nabla p \otimes \nabla u, \end{aligned}$$

12 $$\begin{aligned} {\mathcal {S}}_0^\varOmega&= -2 {\tilde{\alpha }}_\varOmega (u-{\hat{u}}) \nabla {\hat{u}} \end{aligned}$$

where $I \in {\mathbb {R}}^{d,d}$ denotes the identity matrix, and

$$\begin{aligned} L =&[({\mathcal {S}}_1^{\varOmega ,\text {in}} - {\mathcal {S}}_1^{\varOmega ,\text {out}}) n ]\cdot n \\ =&c_1 + c_2 ({\tilde{\alpha }}_1- {\tilde{\alpha }}_2) (u-{\hat{u}})^2 + (\alpha _1-\alpha _2) u p - (f_1 - f_2) p \\&+ (\lambda _1- \lambda _2) (\nabla u \cdot \tau )(\nabla p \cdot \tau ) - \left( \frac{1}{\lambda _1} - \frac{1}{\lambda _2}\right) ( \lambda _\varOmega \nabla u \cdot n)( \lambda _\varOmega \nabla p \cdot n). \end{aligned}$$

Here, ${\mathcal {S}}_1^{\varOmega ,\text {in}}$ and $\mathcal S_1^{\varOmega ,\text {out}}$ denote the restrictions of the tensor ${\mathcal {S}}_1^{\varOmega }$ to $\varOmega$ and $D {\setminus } \varOmega$, respectively. Furthermore, for two column vectors $a, b \in \mathbb R^d$, $a \otimes b = a b^\top \in {\mathbb {R}}^{d \times d}$ denotes their outer product, $\tau$ denotes the tangential vector and $p \in H^1_0(D)$ is the solution to the adjoint equation

$$\begin{aligned} \int _D \lambda _\varOmega \nabla v \cdot \nabla p + \alpha _\varOmega v p \; \text{ d }x = - 2c_2 \int _D {\tilde{\alpha }}_\varOmega (u - {\hat{u}})v \; \text{ d }x \quad \text{ for } \text{ all } v \in H^1_0(D). \end{aligned}$$

Moreover, motivated by the definition of the topological derivative (4) with the volume of the difference of the perturbed and unperturbed domains in the denominator, we introduce the alternative definition of a shape derivative

13 $$\begin{aligned} \hat{d}_S{\mathfrak {g}}(\varOmega )(V) = \underset{t \searrow 0}{\text{ lim } } \frac{{\mathfrak {g}}(\varOmega _t)-{\mathfrak {g}}(\varOmega )}{|\varOmega _t \triangle \varOmega |}, \end{aligned}$$

with the symmetric difference of two sets $A \triangle B:= (A {\setminus } {\overline{B}}) \cup (B {\setminus } {\overline{A}})$. Note that the volume of the symmetric difference in (13) can be written as

14 $$\begin{aligned} |\varOmega _t \triangle \varOmega | = \int _D |\chi _{\varOmega _t} - \chi _{\varOmega }| \;\textrm{d} {{\textbf{x}}}. \end{aligned}$$

#### Lemma 1

Let $\varOmega$ and *V* smooth. It holds

$$\begin{aligned} \underset{t \searrow 0}{\text{ lim } }\frac{1}{t} \left|\varOmega _t \triangle \varOmega \right|= \int _{\partial \varOmega } |V \cdot n| \; \text{ d }S_x. \end{aligned}$$

The proof is given in Appendix A.1. From Lemma 1, we immediately obtain the following relation between $d_S{\mathfrak {g}}(\varOmega )(V)$ and $\hat{d}_S{\mathfrak {g}}(\varOmega )(V)$.

#### Corollary 1

Suppose that ${\mathfrak {g}}$ is shape differentiable at $\varOmega$ and that $\varOmega$ and *V* are smooth and $\int _{\partial \varOmega } |V \cdot n| \; dS_x > 0$. Then it holds

15 $$\begin{aligned} \hat{d}_S{\mathfrak {g}}(\varOmega )(V) = \frac{ d_S{\mathfrak {g}}(\varOmega )(V)}{ \int _{\partial \varOmega } |V \cdot n| \; dS_x}. \end{aligned}$$

#### Proof

This follows immediately from the definition of $\hat{d}_S{\mathfrak {g}}(\varOmega )(V)$ by Lemma 1 since

16 $$\begin{aligned} \hat{d}_S{\mathfrak {g}}(\varOmega )(V) = \underset{t \searrow 0}{\text{ lim } }\frac{{\mathfrak {g}}(\varOmega _t)-{\mathfrak {g}}(\varOmega )}{|\varOmega _t \triangle \varOmega |} = \frac{\underset{t \searrow 0}{\text{ lim } }\frac{{\mathfrak {g}}(\varOmega _t)-{\mathfrak {g}}(\varOmega )}{t}}{\underset{t \searrow 0}{\text{ lim } }\frac{|\varOmega _t \triangle \varOmega |}{t}} = \frac{ d_S{\mathfrak {g}}(\varOmega )(V)}{\int _{\partial \varOmega } |V \cdot n| \; dS_x}. \end{aligned}$$

$$\square $$

#### Remark 1

The condition $\int _{\varOmega } \text{ div }(V) \; dx \ne 0$ is a sufficient condition for $\int _{\partial \varOmega } |V \cdot n| \; dS_x > 0$, since

17 $$\begin{aligned} 0< \left| \int _{\varOmega } \text{ div }(V) \; dx \right| = \left| \int _{\partial \varOmega } V \cdot n \; dS_x \right| < \int _{\partial \varOmega } |V \cdot n| \; dS_x. \end{aligned}$$

### The Continuous Topological-Shape Derivative

Here and in the following, we assume that the domain $\varOmega$ is described by a level-set function $\phi : D \rightarrow {\mathbb {R}}$ via

18a $$\begin{aligned} \phi ({{\textbf{x}}}) < 0&\Longleftrightarrow {{\textbf{x}}}\in \varOmega , \end{aligned}$$

18b $$\begin{aligned} \phi ({{\textbf{x}}}) > 0&\Longleftrightarrow {{\textbf{x}}}\in D \setminus \overline{\varOmega } \end{aligned}$$

18c $$\begin{aligned} \phi ({{\textbf{x}}}) = 0&\Longleftrightarrow {{\textbf{x}}}\in \partial \varOmega \cap D. \end{aligned}$$

For given $\phi$, let $\varOmega (\phi )$ denote the unique domain defined by (18a)–(18c). In this section, in contrast to the setting in Sect. 2.2, we perturb $\varOmega$ indirectly by perturbing $\phi$ such that $\phi _\varepsilon = O_\varepsilon \phi$ for some operator $O_\varepsilon : C^0(D)\rightarrow C^0(D)$ depending on $\varepsilon \ge 0$ with the property $\varOmega (O_0 \phi ) = \varOmega (\phi )$. Later on, in the discrete setting, we will distinguish between two different types of perturbation operators $O_\varepsilon$ corresponding to shape or topological perturbations of $\varOmega$.

Let, from now on, ${\mathcal {J}}(\phi ):= {\mathfrak {g}}(\varOmega (\phi ))$ denote the reduced cost function as a function of the level set function $\phi$. This way, a continuous topological-shape derivative can be defined as

19 $$\begin{aligned} d{\mathcal {J}}(\phi ) = \lim _{\varepsilon \searrow 0}\frac{{\mathcal {J}}(\phi _\varepsilon )-{\mathcal {J}}(\phi )}{|\varOmega (\phi _\varepsilon ) \triangle \varOmega (\phi )|}. \end{aligned}$$

Note that this sensitivity depends on the choice of the perturbation operator $O_\varepsilon$, which can represent either a shape perturbation or a topological perturbation. We will mostly be concerned with its discrete counterpart, which will be introduced in Sect. 4. Note that, in the case of shape perturbations, due to the scaling $|\varOmega (\phi _\varepsilon )\triangle \varOmega (\phi )|$ instead of $\varepsilon$ in the denominator the shape derivative is modified and does not correspond to (8) but rather to (13).

*Relation to literature*

The sensitivity of shape functions with respect to perturbations of a level set function (representing a shape) was investigated in [22] for the case without PDE constraints. There, the author considers smooth level set functions and rigorously computes the Gâteaux (semi-)derivative in the direction of a smooth perturbation of the level set function, both for the case of shape and topological perturbations. In the case of shape perturbations, it is shown that the Gâteaux derivative coincides with the shape derivative (8) with respect to a suitably chosen vector field. On the other hand, a resemblance between the notions of Gâteaux derivative and topological derivative is shown, yet the Gâteaux derivative may vanish or not exist in cases where the topological derivative is finite. Evidently, this discrepancy results from the fact that the denominator in the definition of the Gâteaux derivative is always of order one whereas it is of the order of the space dimension in the topological derivative.

While the analysis for shape and topological perturbations is carried out separately in [22], a more unified approach is followed in [13, 14]. In these publications, the idea is to consider sensitivities with respect to domain perturbations that are obtained by the dilation of lower-dimensional objects. Here, given a set $E \subset {\mathbb {R}}^d$ of dimension $k \le d$, the dilated set of radius *r* is given by $E_r = \{x \in {\mathbb {R}}^d: d_E(x) \le r\}$ where $d_E(x)$ denotes a distance of a point *x* to a set *E*. For instance, when *E* is chosen as a single point, the dilated set is just a ball of radius *r* around that point and performing a sensitivity analysis with respect to the volume of the dilated object leads to the topological derivative. On the other hand, when *E* is chosen as the boundary of a domain, $E_r$ can be defined using a signed distance function and corresponds to a uniform expansion of the domain. Then, a similar procedure leads to the shape derivative with respect to a uniform expansion in normal direction (i.e. $V = n$ in (7)–(8)). In [13], a sensitivity analysis for various choices of *E* is carried out with respect to the volume of the perturbation. We note, however, that arbitrary shape perturbations are not covered and would require an extension of the theory. Comparing [22] and [13], we observe that in the former paper only smooth perturbations of a level set function are admissible whereas, in the latter approach, domain perturbations by dilations can be interpreted as perturbations of level set functions by a (non-smooth) distance function.

Finally, we mention [9] where a domain is represented by a discretized level set function and a shape sensitivity analysis is carried out with respect to a perturbation of the level set values close to the boundary. This procedure can be interpreted as an application of the idea of dilation to discretized shape optimization problems. As the authors point out, this kind of shape sensitivity analysis is more natural compared to the standard approach based on domain transformations when employed in a level-set framework; an observation also made in [22, Sec. 3]. The authors show numerical results for the shape optimization of an acoustic horn, but do not consider topological perturbations in this work.

As it can be seen from (19), our approach is related to the dilation concept since we also consider the sensitivity with respect to the volume of the domain perturbation $\varOmega _t \triangle \varOmega$. In the following, we will investigate the topological-shape derivative in a discretized setting. Similarly to [9], we will consider shape sensitivity analysis with respect to level set values on mesh nodes close to the boundary. Moreover, we will also be able to deal with topological perturbations and treat shape and topological updates in a unified way by a discretized version of (19), called the numerical topological-shape derivative.

#### Remark 2

The concept of the topological-shape derivative which we introduce in this paper should not be confused with the topological-shape sensitivity method introduced in [27, 28]. While the new concept represents a unified sensitivity with respect to both shape and topological perturbations, the sensitivity method [27, 28] is a method to compute the pointwise topological derivative as a limit of a certain shape derivative and thus does not account for shape changes at physical material interfaces.

## Numerical Setting

In this section we consider the discretization of (2). Let $\mathcal {T}$ be a given finite element mesh covering *D* with *M* nodes $\{{{\textbf{x}}}_k\}_{k=1}^{M}$ and *N* triangular elements $\{\tau _l\}_{l=1}^N$. We introduce the index set $I_{{{\textbf{x}}}_k}$ of all element indices of elements $\tau _l$ where ${{\textbf{x}}}_k$ is a node of $\tau _l$,

20 $$\begin{aligned} I_{{{\textbf{x}}}_k}:= \{l \in {\{1, \dots , N \} }: {{\textbf{x}}}_k \in {\bar{\tau }}_l\} \quad \text {for } k = 1,\dots ,M. \end{aligned}$$

Moreover,

21 $$\begin{aligned} J_{\tau _l}:= \{k \in {\{1, \dots , M \} }: {{\textbf{x}}}_k \in {\bar{\tau }}_l\} \quad \text {for } l = 1,\dots ,N \end{aligned}$$

is the index set of all node indices of nodes ${{\textbf{x}}}_k$ in $\bar{\tau }_l$. Furthermore, we introduce the one-ring of a node ${{\textbf{x}}}_k$,

22 $$\begin{aligned} R_{{{\textbf{x}}}_k}:= \{i \in {\{1, \dots , M \} } |\exists l \in I_{{{\textbf{x}}}_k}: {{\textbf{x}}}_i \in {\bar{\tau }}_l\} \quad \text {for } k = 1,\dots ,M. \end{aligned}$$

**Fig. 1:**
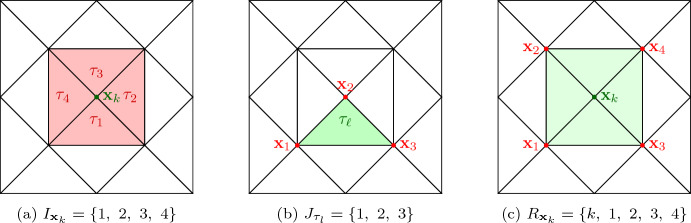
Illustration of the sets $I_{{{\textbf{x}}}_k}$, $J_{\tau _l}$, and $R_{{{\textbf{x}}}_k}$

These sets are illustrated in Fig. 1.

Let $P_1 =\{a+bx_1 + cx_2: a,b,c \in {\mathbb {R}}\}$ denote the space of affine linear polynomials in two space dimensions and $S_h^1(D)$ the space of piecewise affine linear and globally continuous functions on $\mathcal {T}$,

$$\begin{aligned} S_h^1(D) = \{ v \in H^1(D): v|_{T} \in P_1 \text{ for } \text{ all } T \in \mathcal {T}\} = \text{ span } \{ \varphi _1, \dots , \varphi _M \} \end{aligned}$$

with the hat basis functions $\varphi _i \in S_h^1(D)$ which satisfy $\varphi _i({{\textbf{x}}}_j) = \delta _{ij}$, $i,j = 1, \dots , M$. The discretization of problem (2) leads to the discretized optimization problem

23a $$\begin{aligned} \underset{\varOmega }{\text{ min } }&c_1|\varOmega | + c_2 ({\textbf{u}} - \hat{{\textbf{u}}})^\top \tilde{{\textbf{M}}}({\textbf{u}} - \hat{{\textbf{u}}}) \end{aligned}$$

23b $$\begin{aligned}&\text{ subject } \text{ to } {\textbf{A}} {\textbf{u}} = {\textbf{f}}, \end{aligned}$$

with the solution vector ${\textbf{u}} \in {\mathbb {R}}^{M}$ and ${\textbf{A}} = {\textbf{M}} + {\textbf{K}}$. Here, the mass matrices ${\textbf{M}}, \tilde{{\textbf{M}}} \in {\mathbb {R}}^{M\times M}$, the stiffness matrix $\textbf{K}\in {\mathbb {R}}^{M\times M}$, and the right-hand-side vector ${\textbf{f}} \in {\mathbb {R}}^{M}$ depend on the shape $\varOmega$ and are given by

24 $$\begin{aligned} \begin{aligned} {\textbf{M}}[i,j]&= \int _{D} \alpha _{\varOmega } \varphi _j \varphi _i \;\textrm{d} {{\textbf{x}}},{} & {} {}&{\textbf{K}}[i,j]&= \int _{D} \lambda _{\varOmega } \nabla \varphi _j \cdot \nabla \varphi _i \;\textrm{d} {{\textbf{x}}}, \\ {\textbf{f}}[i]&= \int _{D} f_{\varOmega }\varphi _i \, \;\textrm{d} {{\textbf{x}}},{} & {} {}&\tilde{{\textbf{M}}}[i,j]&= \int _{D} {\tilde{\alpha }}_{\varOmega }\varphi _j \varphi _i \;\textrm{d} {{\textbf{x}}}. \end{aligned} \end{aligned}$$

On the reference element $\tau _R = \{\xi \in {\mathbb {R}}^2:0\le \xi _1\le 1,0\le \xi _2\le 1-\xi _1\}$ we have the local form functions

$$\begin{aligned} \psi _1(\xi _1, \xi _2)&= 1 -\xi _1 -\xi _2,{} & {} {}&\psi _2(\xi _1, \xi _2)&= \xi _1,{} & {} {}&\psi _3(\xi _1, \xi _2)&= \xi _2. \end{aligned}$$

For an element $\tau _l \in \mathcal {T}$, we denote the global vertex indices of its three vertices by $l_1$, $l_2$, $l_3$ and assume them to be numbered in counter-clockwise orientation. Then, the respective local finite element matrices and the local right-hand-side vector for element $\tau _l$ are given by

$$\begin{aligned} {\textbf{m}}_l[i,j]&= \left| {\text {det} }J_l\right| \int _{\xi _1=0}^1\int _{\xi _2=0}^{1-\xi _1} {(\alpha _{\varOmega } \circ \Phi _l)} \psi _j \psi _i \;\textrm{d} \xi _2 \;\textrm{d} \xi _1, \\ {\textbf{k}}_l[i,j]&= \left| {\text {det} }J_l\right| \left( J_l^{-1}\nabla _\xi \psi _j\right) ^\top \left( J_l^{-1}\nabla _\xi \psi _i\right) \int _{\xi _1=0}^1\int _{\xi _2=0}^{1-\xi _1} {(\lambda _{\varOmega } \circ \Phi _l)} \;\textrm{d} \xi _2 \;\textrm{d} \xi _1, \\ {\textbf{f}}_l[i]&= \left| {\text {det} }J_l\right| \int _{\xi _1=0}^1\int _{\xi _2=0}^{1-\xi _1} {(f_{\varOmega } \circ \Phi _l)} \, \psi _i \;\textrm{d} \xi _2 \;\textrm{d} \xi _1, \\ \end{aligned}$$

for $i, j \in \{1,2,3\}$, where $\nabla _\xi \psi _j = [\partial _{\xi _1}\psi _j, \partial _{\xi _2}\psi _j]^\top$ and the mapping $\Phi _l$ between $\tau _R$ and $\tau _l$ and its Jacobian $J_l$ are given by

$$\begin{aligned} \Phi _l(\xi _1, \xi _2) = {{\textbf{x}}}_{l_1} + J_l \begin{bmatrix}\xi _1 \\ \xi _2\end{bmatrix}, \qquad J_l = \begin{bmatrix} {{\textbf{x}}}_{l_2}-{{\textbf{x}}}_{l_1}&{{\textbf{x}}}_{l_3}-{{\textbf{x}}}_{l_1} \end{bmatrix} \in {\mathbb {R}}^{2 \times 2}. \end{aligned}$$

## Numerical Topological-Shape Derivative

Given the discretization introduced in Sect. 3, in contrast to the continuous topological-shape derivative, the numerical topological-shape derivative is only defined at the nodes of the finite element mesh $\mathcal {T}$. For a given piecewise linear level set function $\phi \in S_h^1(D)$ let $\varOmega (\phi )$ be defined by (18a)–(18c) and ${\mathcal {J}}(\phi ) = g(\varOmega (\phi ),u(\phi ))$, where $u(\phi ) \in {V_h} = \{v \in S_h^1(D): v = g_D \text{ on } \Gamma _D \}$ is the finite element function corresponding to the solution of (23b). Note that, in this section, $\varOmega (\phi )$ is polygonal since $\phi \in S_h^1(D)$. The topological-shape derivative at node ${{\textbf{x}}}_k \in \mathcal {T}$ is defined by

25 $$\begin{aligned} d{\mathcal {J}}(\phi )({{\textbf{x}}}_k) = {\left\{ \begin{array}{ll} \lim _{\varepsilon \searrow 0} \frac{{\mathcal {J}}({T^{-\rightarrow +}_{k,\varepsilon }} \phi )-{\mathcal {J}}(\phi )}{|\varOmega ({T^{-\rightarrow +}_{k,\varepsilon }}\phi ) \triangle \varOmega (\phi )|} \quad \text {for } {{\textbf{x}}}_k \in {\mathfrak {T}}^-(\phi ), \\ \lim _{\varepsilon \searrow 0} \frac{{\mathcal {J}}({T^{+\rightarrow -}_{k,\varepsilon }} \phi )-{\mathcal {J}}( \phi )}{|\varOmega ({T^{+\rightarrow -}_{k,\varepsilon }} \phi ) \triangle \varOmega (\phi )|} \quad \text {for } {{\textbf{x}}}_k \in {\mathfrak {T}}^+(\phi ), \\ \lim _{\varepsilon \searrow 0}\frac{{\mathcal {J}}(S_{k,\varepsilon } \phi )-{\mathcal {J}}(\phi )}{|\varOmega (S_{k,\varepsilon } \phi ) \triangle \varOmega ( \phi )|} \quad \text {for } {{\textbf{x}}}_k \in {\mathfrak {S}}(\phi ). \end{array}\right. } \end{aligned}$$

Here, given $\phi \in S_h^1(D)$, the respective sets are defined by

26a $$\begin{aligned} {\mathfrak {T}}^-(\phi ) =&\{{{\textbf{x}}}_k \in \mathcal {T}\, | \, \forall i \in R_{{{\textbf{x}}}_k}:\phi ({{\textbf{x}}}_i) \le 0 \}, \end{aligned}$$

26b $$\begin{aligned} {\mathfrak {T}}^+(\phi ) =&\{{{\textbf{x}}}_k \in \mathcal {T}\, |\, \forall i \in R_{{{\textbf{x}}}_k}:\phi ({{\textbf{x}}}_i) \ge 0 \}, \end{aligned}$$

26c $$\begin{aligned} {\mathfrak {S}}(\phi ) =&\mathcal {T}\setminus ({\mathfrak {T}}^-(\phi ) \cup {\mathfrak {T}}^+(\phi )). \end{aligned}$$

**Fig. 2:**
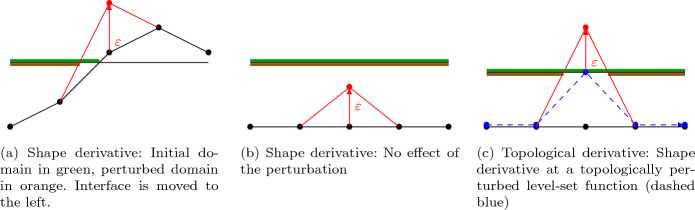
Illustration of the shape derivative and the topological derivative

Whenever, the level set function $\phi$ is clear from the context, we will drop the argument and write ${\mathfrak {T}}^-, {\mathfrak {T}}^+, {\mathfrak {S}}$ for brevity. Furthermore, $T^{-\rightarrow +}_{k,\varepsilon }: S_h^1(D) \rightarrow S_h^1(D)$ is the positive discrete topological perturbation operator defined by its action

27 $$\begin{aligned} T^{-\rightarrow +}_{k,\varepsilon }\phi ({{\textbf{x}}}) = \phi ({{\textbf{x}}}) -( \phi ({{\textbf{x}}}_k) - \varepsilon ) \varphi _k({{\textbf{x}}}), \end{aligned}$$

see Fig. 2(c), whereas the negative discrete topological perturbation operator $T^{+\rightarrow -}_{k,\varepsilon }: S_h^1(D) \rightarrow S_h^1(D)$ is defined by

28 $$\begin{aligned} T^{+\rightarrow -}_{k,\varepsilon }\phi ({{\textbf{x}}}) = \phi ({{\textbf{x}}}) -( \phi ({{\textbf{x}}}_k) + \varepsilon ) \varphi _k({{\textbf{x}}}). \end{aligned}$$

Finally, the discrete shape perturbation operator $S_{k,\varepsilon }: S_h^1(D) \rightarrow S_h^1(D)$ is defined by

29 $$\begin{aligned} S_{k,\varepsilon }\phi ({{\textbf{x}}}) := \phi ({{\textbf{x}}}) + \varepsilon \varphi _k({{\textbf{x}}}). \end{aligned}$$

### Remark 3

Note that the discrete perturbation operators defined above only change the nodal value of the finite element function $\phi \in S_h^1(D)$ at one node ${{\textbf{x}}}_k$, *e.g.* for $S_{k,\varepsilon }$ it holds $S_{k,\varepsilon }\phi ({{\textbf{x}}}_j) = \phi ({{\textbf{x}}}_j)$ for all $j \in \mathcal {T}{\setminus } \{k\}$ and $S_{k,\varepsilon }\phi ({{\textbf{x}}}_k) = \phi ({{\textbf{x}}}_k) + \varepsilon$.

### Computation of the Numerical Topological-Shape Derivative for the Area Cost Functional

Before we compute the numerical topological-shape derivative (25) for the full model problem (2), we consider the case of the pure volume cost function and neglect the PDE constraint, i.e., we set $c_1 = 1, c_2 =0$ in (1).

**Fig. 3:**
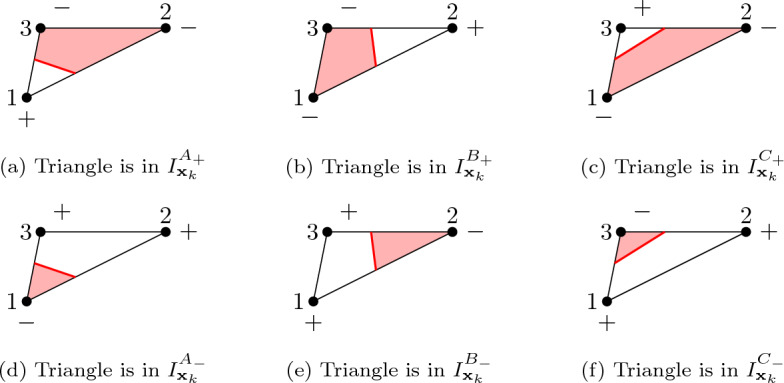
Illustration of the sets $I_{{{\textbf{x}}}_k}^{A_+},~I_{{{\textbf{x}}}_k}^{A_-},~I_{{{\textbf{x}}}_k}^{B_+},~I_{{{\textbf{x}}}_k}^{B_-},~I_{{{\textbf{x}}}_k}^{C_+},~ I_{{{\textbf{x}}}_k}^{C_-}$. The nodal values of the level-set functions are indicated by −, $+$. The interface is drawn in red

For that purpose, we investigate

30 $$\begin{aligned} \delta _{k,\varepsilon } a= |\varOmega (O_{k,\varepsilon }\phi )|-|\varOmega (\phi )|, \end{aligned}$$

for $O_{k,\varepsilon }\in \{ T^{-\rightarrow +}_{k,\varepsilon },\,T^{+\rightarrow -}_{k,\varepsilon },\,S_{k,\varepsilon }\}$. We note that for the computation of (30) only those "cut elements" are relevant which have a node ${{\textbf{x}}}_k$, *i.e.* 

31 $$\begin{aligned} \delta _{k,\varepsilon } a= {\sum _{l\in C_k} \delta _{k,\varepsilon }a_l} \quad \text{ with } \delta _{k,\varepsilon }a_l:= \int _{\tau _l} H(-O_{k,\varepsilon }\phi ) - H(-\phi ) \;\textrm{d} {{\textbf{x}}}, \end{aligned}$$

where *H*(*x*) denotes the Heaviside step function and

32 $$\begin{aligned} C_k = \{l \in I_{{{\textbf{x}}}_k}: \tau _l \cap \partial \varOmega (O_{k,\varepsilon }\phi ) \ne \emptyset \} \end{aligned}$$

is the set of all indices of elements adjacent to ${{\textbf{x}}}_k$ which are intersected by the perturbed interface. Note that, for $\varepsilon >0$ small enough, $C_k$ does not depend on the concrete value of $\varepsilon$. For an element $\tau _l$ with $l \in I_{{{\textbf{x}}}_k}$ we denote the three vertices in counter-clockwise orientation by ${{\textbf{x}}}_{l_1}$, ${{\textbf{x}}}_{l_2}$, ${{\textbf{x}}}_{l_3}$ and assume that ${{\textbf{x}}}_k = {{\textbf{x}}}_{l_1}$. Moreover we denote $\phi _{l_j}:= \phi ({{\textbf{x}}}_{l_j})$ and ${\tilde{\phi }}_{l_j}:= O_{k,\varepsilon }\phi ({{\textbf{x}}}_{l_j})$ for $j=1,2,3$ and small enough $\varepsilon$. In the following, we will be interested in

33 $$\begin{aligned} d_k a := \sum _{l \in C_k} d_k a_l \quad \text{ with } d_k a_l := \underset{\varepsilon \searrow 0}{\text{ lim } } \frac{\delta _{k,\varepsilon } a_l}{\varepsilon ^o}. \end{aligned}$$

with $\delta _{k,\varepsilon } a_l$ defined in (30). Here, $o=1$ in the case of a shape perturbation and $o=2$ in the case of a topological perturbation. We consider six different sets (see Fig. 3 for an illustration)

34 $$\begin{aligned} \begin{aligned} I_{{{\textbf{x}}}_k}^{A_+} =&\{ l \in I_{{{\textbf{x}}}_k}: {\tilde{\phi }}_{l_1}> 0, {\tilde{\phi }}_{l_2}< 0, {\tilde{\phi }}_{l_3}< 0 \},{} & {} {}&I_{{{\textbf{x}}}_k}^{A_-} =&\{ l \in I_{{{\textbf{x}}}_k}: {\tilde{\phi }}_{l_1}< 0, {\tilde{\phi }}_{l_2}> 0, {\tilde{\phi }}_{l_3}> 0 \}, \\ I_{{{\textbf{x}}}_k}^{B_+} =&\{ l \in I_{{{\textbf{x}}}_k}: {\tilde{\phi }}_{l_1}< 0, {\tilde{\phi }}_{l_2}> 0, {\tilde{\phi }}_{l_3}< 0 \},{} & {} {}&I_{{{\textbf{x}}}_k}^{B_-} =&\{ l \in I_{{{\textbf{x}}}_k}: {\tilde{\phi }}_{l_1}> 0, {\tilde{\phi }}_{l_2}< 0, {\tilde{\phi }}_{l_3}> 0 \}, \\ I_{{{\textbf{x}}}_k}^{C_+} =&\{ l \in I_{{{\textbf{x}}}_k}: {\tilde{\phi }}_{l_1}< 0, {\tilde{\phi }}_{l_2}< 0, {\tilde{\phi }}_{l_3}> 0 \},{} & {} {}&I_{{{\textbf{x}}}_k}^{C_-} =&\{ l \in I_{{{\textbf{x}}}_k}: {\tilde{\phi }}_{l_1}> 0, {\tilde{\phi }}_{l_2} > 0, {\tilde{\phi }}_{l_3} < 0 \}, \end{aligned} \end{aligned}$$

such that

$$\begin{aligned} C_k = I_{{{\textbf{x}}}_k}^{A_+} \cup I_{{{\textbf{x}}}_k}^{A_-} \cup I_{{{\textbf{x}}}_k}^{B_+} \cup I_{{{\textbf{x}}}_k}^{B_-} \cup I_{{{\textbf{x}}}_k}^{C_+} \cup I_{{{\textbf{x}}}_k}^{C_-} \end{aligned}$$

with a direct sum on the right hand side. We can thus split the sum in (31) into six parts,

$$\begin{aligned} \delta _{k,\varepsilon } a= \mathcal {I}_{I_{{{\textbf{x}}}_k}}^{A_+} + \mathcal {I}_{I_{{{\textbf{x}}}_k}}^{A_-} + \mathcal {I}_{I_{{{\textbf{x}}}_k}}^{B_+} + \mathcal {I}_{I_{{{\textbf{x}}}_k}}^{B_-} + \mathcal {I}_{I_{{{\textbf{x}}}_k}}^{C_+} + \mathcal {I}_{I_{{{\textbf{x}}}_k}}^{C_-} . \end{aligned}$$

**Fig. 4:**
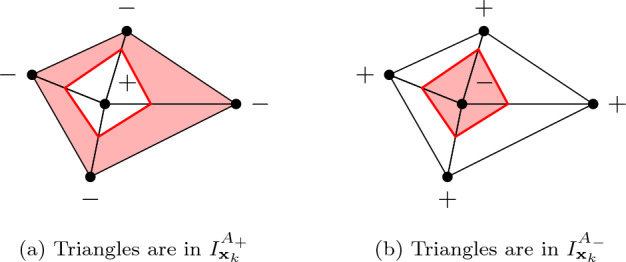
Illustration of $I_{{{\textbf{x}}}_k}^{A_+}$ and $I_{{{\textbf{x}}}_k}^{A_-}$ in the case of topological perturbations

*Configuration A* For $l \in I_{{{\textbf{x}}}_k}^{A_+}$ we have

35 $$\begin{aligned} \int _{\tau _{l}} H(-O_{k,\varepsilon }\phi ({{\textbf{x}}})) \;\textrm{d} {{\textbf{x}}}= & {} \frac{\left| {\text {det} }J_l\right| }{2} \left( 1- \int _{\xi _1=0}^{\ell _1} \int _{\xi _2=0}^{\ell _2\left( 1-\frac{\xi _1}{\ell _1}\right) } \,d\xi _2d\xi _1 \right) \nonumber \\= & {} \frac{\left| {\text {det} }J_l\right| }{2}(1-\ell _1\ell _2), \end{aligned}$$

with

$$\begin{aligned} \ell _1 = \frac{\phi _{l_1}+\varepsilon }{\phi _{l_1}+\varepsilon -\phi _{l_2}}, \qquad \ell _2 = \frac{\phi _{l_1}+\varepsilon }{\phi _{l_1}+\varepsilon -\phi _{l_3}}. \end{aligned}$$

Therefore,

36 $$\begin{aligned} \mathcal {I}_{I_{{{\textbf{x}}}_k}}^{A_+}= & {} \sum _{l \in I_{{{\textbf{x}}}_k}^{A_+}} \frac{\left| {\text {det} }J_l\right| }{2} \left( -\frac{(\phi _{l_1}+\varepsilon )^2}{(\phi _{l_1}+\varepsilon -\phi _{l_2})(\phi _{l_1}+\varepsilon -\phi _{l_3})}+\frac{\phi _{l_1}^2}{(\phi _{l_1}-\phi _{l_2})(\phi _{l_1}-\phi _{l_3})} \right) \nonumber \\= & {} \sum _{l \in I_{{{\textbf{x}}}_k}^{A_+}} \frac{\left| {\text {det} }J_l\right| }{2} \left( \frac{\varepsilon \phi _{l_1} \left[ \phi _{l_1}(\phi _{l_2} + \phi _{l_3})-2\phi _{l_2}\phi _{l_3} \right] +\varepsilon ^2\left[ \phi _{l_1}(\phi _{l_2} + \phi _{l_3})-\phi _{l_2}\phi _{l_3} \right] }{(\phi _{l_1} - \phi _{l_2})^2(\phi _{l_1} - \phi _{l_3})^2 + O(\varepsilon )} \right) . \nonumber \\ \end{aligned}$$

For ${{\textbf{x}}}_k \in {\mathfrak {T}}^-$ and $O_{k,\varepsilon }= T^{-\rightarrow +}_{k,\varepsilon }$, it holds $I_{{{\textbf{x}}}_k}=I_{{{\textbf{x}}}_k}^{A_+}$ (see Fig. 4a) and we have to consider (36) with $\phi _{l_1} = 0$. Thus, we obtain

37 $$\begin{aligned} \mathcal {I}_{I_{{{\textbf{x}}}_k}}^{A_+} = - \sum _{l \in I_{{{\textbf{x}}}_k}^{A_+}}\frac{\varepsilon ^2\left| {\text {det} }J_l\right| }{2} \frac{\phi _{l_2}\phi _{l_3} }{ \phi _{l_2}^2\phi _{l_3}^2 + O(\varepsilon )}, \end{aligned}$$

and conclude for this case

38 $$\begin{aligned} d_{k}a = \lim _{\varepsilon \searrow 0}\frac{\delta _{k,\varepsilon } a}{\varepsilon ^2} =- \sum _{l \in I_{{{\textbf{x}}}_k}^{A_+}}\frac{\left| {\text {det} }J_l\right| }{2\phi _{l_2}\phi _{l_3}}. \end{aligned}$$

Moreover, for ${{\textbf{x}}}_k \in {\mathfrak {T}}^+$ and $O_{k,\varepsilon }= T^{+\rightarrow -}_{k,\varepsilon }$, it holds $I_{{{\textbf{x}}}_k} = I_{{{\textbf{x}}}_k}^{A_-}$ (see Fig. 4b) and we have for $l \in I_{{{\textbf{x}}}_k}^{A_-}$,

$$\begin{aligned} \int _{\tau _{l}} H(-O_{k,\varepsilon }\phi ({{\textbf{x}}})) \;\textrm{d} {{\textbf{x}}}&= \frac{\left| {\text {det} }J_l\right| }{2} \int _{\xi _1=0}^{\ell _1} \int _{\xi _2=0}^{\ell _2\left( 1-\frac{\xi _1}{\ell _1}\right) } \,d\xi _2d\xi _1 = \frac{\left| {\text {det} }J_l\right| }{2}\ell _1\ell _2, \end{aligned}$$

which leads to

39 $$\begin{aligned} \begin{aligned} \mathcal {I}_{I_{{{\textbf{x}}}_k}}^{A_-}&= -\sum _{l \in I_{{{\textbf{x}}}_k}^{A_-}} \frac{\left| {\text {det} }J_l\right| }{2} \left( \frac{\varepsilon \phi _{l_1} \left[ \phi _{l_1}(\phi _{l_2} + \phi _{l_3})-2\phi _{l_2}\phi _{l_3} \right] +\varepsilon ^2\left[ \phi _{l_1}(\phi _{l_2} + \phi _{l_3})-\phi _{l_2}\phi _{l_3} \right] }{(\phi _{l_1} - \phi _{l_2})^2(\phi _{l_1} - \phi _{l_3})^2 + O(\varepsilon )} \right) . \end{aligned} \end{aligned}$$

Therefore, we obtain for this case

$$\begin{aligned} d_{k}a = \lim _{\varepsilon \searrow 0}\frac{\delta _{k,\varepsilon } a}{\varepsilon ^2} = \sum _{l \in I_{{{\textbf{x}}}_k}^{A_-}}\frac{\left| {\text {det} }J_l\right| }{2\phi _{l_2}\phi _{l_3}}. \end{aligned}$$

Finally, for ${{\textbf{x}}}_k \in {\mathfrak {S}}$ and $O_{k,\varepsilon }= S_{k,\varepsilon }$ we deduce from (36)

40 $$\begin{aligned} \lim _{\varepsilon \searrow 0}\frac{\mathcal {I}_{I_{{{\textbf{x}}}_k}}^{A_+} }{\varepsilon } = \sum _{l \in I_{{{\textbf{x}}}_k}^{A_+}}\frac{\left| {\text {det} }J_l\right| \;\phi _{l_1} \left[ \phi _{l_1}(\phi _{l_2} + \phi _{l_3}) - 2\phi _{l_2}\phi _{l_3} \right] }{2(\phi _{l_1} - \phi _{l_2})^2(\phi _{l_1} - \phi _{l_3})^2}, \end{aligned}$$

and from (39)

41 $$\begin{aligned} \lim _{\varepsilon \searrow 0}\frac{\mathcal {I}_{I_{{{\textbf{x}}}_k}}^{A_-} }{\varepsilon } = -\sum _{l \in I_{{{\textbf{x}}}_k}^{A_-}}\frac{\left| {\text {det} }J_l\right| \;\phi _{l_1} \left[ \phi _{l_1}(\phi _{l_2} + \phi _{l_3})-2\phi _{l_2}\phi _{l_3} \right] }{2(\phi _{l_1} - \phi _{l_2})^2(\phi _{l_1} - \phi _{l_3})^2}. \end{aligned}$$

*Configuration B* We note that configuration B can only occur for the case ${{\textbf{x}}}_k \in {\mathfrak {S}}$ and $O_{k,\varepsilon }= S_{k,\varepsilon }$. For $l \in I_{{{\textbf{x}}}_k}^{B_+}$ it holds

42 $$\begin{aligned} \int _{\tau _{l}} H(-S_{k,\varepsilon }\phi ({{\textbf{x}}})) \;\textrm{d} {{\textbf{x}}}= \frac{\left| {\text {det} }J_l\right| }{2}\left( 1- \frac{\phi _{l_2}^2}{(\phi _{l_2}-\phi _{l_3})(\phi _{l_2}-\phi _{l_1}-\varepsilon )} \right) , \end{aligned}$$

and

$$\begin{aligned} \begin{aligned} \mathcal {I}_{I_{{{\textbf{x}}}_k}}^{B_+}&= \sum _{l \in I_{{{\textbf{x}}}_k}^{B_+}}\frac{\left| {\text {det} }J_l\right| \phi _{l_2}^2}{2} \frac{(\phi _{l_2}-\phi _{l_3})(\phi _{l_2}-\phi _{l_1}-\varepsilon )-(\phi _{l_2}-\phi _{l_3})(\phi _{l_2}-\phi _{l_1})}{(\phi _{l_2}-\phi _{l_3})(\phi _{l_2}-\phi _{l_1})(\phi _{l_2}-\phi _{l_3})(\phi _{l_2}-\phi _{l_1}-\varepsilon )}\\&= \sum _{l \in I_{{{\textbf{x}}}_k}^{B_+}}\frac{\left| {\text {det} }J_l\right| \phi _{l_2}^2}{2} \frac{-\varepsilon (\phi _{l_2}-\phi _{l_3})}{(\phi _{l_2}-\phi _{l_3})^2(\phi _{l_2}-\phi _{l_1})(\phi _{l_2}-\phi _{l_1}-\varepsilon )}. \end{aligned} \end{aligned}$$

Thus,

43 $$\begin{aligned} \lim _{\varepsilon \searrow 0}\frac{\mathcal {I}_{I_{{{\textbf{x}}}_k}}^{B_+} }{\varepsilon } = -\sum _{l \in I_{{{\textbf{x}}}_k}^{B_+}} \frac{\left| {\text {det} }J_l\right| }{2} \frac{\phi _{l_2}^2}{(\phi _{l_2}-\phi _{l_3})(\phi _{l_2}-\phi _{l_1})^2}. \end{aligned}$$

For the case $l \in I_{{{\textbf{x}}}_k}^{B_-}$ we have

44 $$\begin{aligned} \int _{\tau _{l}} H(-S_{k,\varepsilon }\phi ({{\textbf{x}}})) \;\textrm{d} {{\textbf{x}}}= \frac{\left| {\text {det} }J_l\right| }{2} \frac{\phi _{l_2}^2}{(\phi _{l_2}-\phi _{l_3})(\phi _{l_2}-\phi _{l_1}-\varepsilon )}, \end{aligned}$$

and obtain

45 $$\begin{aligned} \lim _{\varepsilon \searrow 0}\frac{\mathcal {I}_{I_{{{\textbf{x}}}_k}}^{B_-} }{\varepsilon } = \sum _{l \in I_{{{\textbf{x}}}_k}^{B_-}} \frac{\left| {\text {det} }J_l\right| }{2} \frac{\phi _{l_2}^2}{(\phi _{l_2}-\phi _{l_3})(\phi _{l_2}-\phi _{l_1})^2}. \end{aligned}$$

*Configuration C* Analogously as for configuration B, we note that configuration C can only occur for the case ${{\textbf{x}}}_k \in {\mathfrak {S}}$ and $O_{k,\varepsilon }= S_{k,\varepsilon }$. Furthermore, the formulas for configuration C can be obtained by exchanging $l_3$ and $l_2$ in the formulas (43) and (45).

Summarizing, we have shown the following result.

#### Theorem 1

For ${{\textbf{x}}}_k \in {\mathfrak {T}}^-$ we have

46 $$\begin{aligned} d_k a = \sum _{l \in I_{{{\textbf{x}}}_k}^{A_+}} d_ka_l = \sum _{l \in I_{{{\textbf{x}}}_k}^{A_+}}(-1)\frac{\left| {\text {det} }J_l\right| }{2\phi _{l_2}\phi _{l_3}}. \end{aligned}$$

For ${{\textbf{x}}}_k \in {\mathfrak {T}}^+$ we have

47 $$\begin{aligned} d_k a = \sum _{l \in I_{{{\textbf{x}}}_k}^{A_-}} d_ka_l = \sum _{l \in I_{{{\textbf{x}}}_k}^{A_-}}\frac{\left| {\text {det} }J_l\right| }{2\phi _{l_2}\phi _{l_3}}. \end{aligned}$$

For ${{\textbf{x}}}_k \in {\mathfrak {S}}$ we have

48 $$\begin{aligned} d_k a= & {} \sum _{l \in I_{{{\textbf{x}}}_k}^{A_+}}\mathcal {I}_0(\phi _{l_1},\phi _{l_2},\phi _{l_3}) - \sum _{l \in I_{{{\textbf{x}}}_k}^{A_-}} \mathcal {I}_0(\phi _{l_1},\phi _{l_2},\phi _{l_3}) -\sum _{l \in I_{{{\textbf{x}}}_k}^{B_+}} \mathcal {I}_1(\phi _{l_1},\phi _{l_2},\phi _{l_3}) \nonumber \\{} & {} + \sum _{l \in I_{{{\textbf{x}}}_k}^{B_-}} \mathcal {I}_1(\phi _{l_1},\phi _{l_2},\phi _{l_3}) -\sum _{l \in I_{{{\textbf{x}}}_k}^{C_+}} \mathcal {I}_1(\phi _{l_1},\phi _{l_3},\phi _{l_2}) + \sum _{l \in I_{{{\textbf{x}}}_k}^{C_-}} \mathcal {I}_1(\phi _{l_1},\phi _{l_3},\phi _{l_2}), \nonumber \\ \end{aligned}$$

with

$$\begin{aligned} \mathcal {I}_0(\phi _{l_1},\phi _{l_2},\phi _{l_3})&= \frac{\left| {\text {det} }J_l\right| \;\phi _{l_1} \left[ \phi _{l_1}(\phi _{l_2} + \phi _{l_3}) - 2\phi _{l_2}\phi _{l_3} \right] }{2(\phi _{l_1} - \phi _{l_2})^2(\phi _{l_1} - \phi _{l_3})^2}, \\ \mathcal {I}_1(\phi _{l_1},\phi _{l_2},\phi _{l_3})&= \frac{\left| {\text {det} }J_l\right| \;\phi _{l_2}^2}{2(\phi _{l_2}-\phi _{l_3})(\phi _{l_2}-\phi _{l_1})^2}. \end{aligned}$$

#### Remark 4

The corresponding computations for the denominators in (25), *i.e.* $|\varOmega (O_{k,\varepsilon }\phi )\triangle \varOmega (\phi )|$, are closely related to the computations presented in this section for (30). Denoting

$$\begin{aligned} \delta _{k,\varepsilon } {\tilde{a}}_l := \int _{\tau _l} | H(-O_{k,\varepsilon }\phi ) - H(-\phi )| \;\textrm{d} {{\textbf{x}}}, \quad d_k {\tilde{a}}_l := \underset{\varepsilon \searrow 0}{\text{ lim }} \frac{\delta _{k,\varepsilon } {\tilde{a}}_l}{\varepsilon ^o}, \end{aligned}$$

we get that

49 $$\begin{aligned} \underset{\varepsilon \searrow 0}{\text{ lim } } \frac{|\varOmega (O_{k,\varepsilon }\phi ) \triangle \varOmega (\phi )|}{\varepsilon ^o} = \sum _{l\in C_k} d_k {\tilde{a}}_l =: d_k {\tilde{a}} \end{aligned}$$

where $d_k {\tilde{a}}_l = |d_k a_l|$ with the formulas for $d_k a_l$ given in (46)–(48). It is obvious that, for ${{\textbf{x}}}_k \in {\mathcal {T}}^-$, $d_k a_l <0$ and for ${{\textbf{x}}}_k \in {\mathcal {T}}^+$, $d_k a_l >0$. Moreover, note that, for ${{\textbf{x}}}_k \in {\mathcal {S}}$, it holds $d_k a_l <0$ for all $l \in C_k$. This yields that

50 $$\begin{aligned} \frac{d_k a_l}{d_k {\tilde{a}}_l} = {\left\{ \begin{array}{ll} - 1 &{} \text{ if } k \in {\mathcal {S}} \cup {\mathcal {T}}^-, \\ \; \;\, 1 &{} \text{ if } k \in {\mathcal {T}}^+. \end{array}\right. } \end{aligned}$$

#### Corollary 2

From Theorem 1 and Remark 4, it follows that the numerical topological-shape derivative of the volume cost function $\text{ Vol }(\phi ):= |\varOmega (\phi )|$ is given by

$$\begin{aligned} d \text{ Vol }(\phi )({{\textbf{x}}}_k) = {\left\{ \begin{array}{ll} - 1 &{} \text{ if } k \in {\mathcal {S}} \cup {\mathcal {T}}^-, \\ \; \;\, 1 &{} \text{ if } k \in {\mathcal {T}}^+. \end{array}\right. } \end{aligned}$$

### Computation of the Numerical Topological-Shape Derivative via Lagrangian Framework

Next, we consider the computation of the numerical topological-shape derivative of an optimization problem that is constrained by a discretized PDE. For that purpose, we set $c_1 =0$ in (1) and $J(\phi ,{\textbf{u}}):= g(\varOmega (\phi ),{\textbf{u}})$. The discretized problem reads

51 $$\begin{aligned} \underset{\phi }{\text{ min } } J(\phi , {\textbf{u}})&\end{aligned}$$

52 $$\begin{aligned} \text{ s.t. } {\textbf{A}}_{\phi } {\textbf{u}}&= {\textbf{f}}_{\phi } \end{aligned}$$

and we are interested in the sensitivity of $J$ when the level set function $\phi$ representing the geometry is replaced by a perturbed level set function $\phi _{\varepsilon }=O_{k,\varepsilon }\phi$. The perturbed Lagrangian for (51)–(52) with respect to a perturbation of $\phi$ reads

53 $$\begin{aligned} L(\varepsilon , {\textbf{u}}, {\textbf{v}}) =&J(\phi _\varepsilon , {\textbf{u}}) + {\textbf{A}}_\varepsilon {\textbf{u}}\cdot {\textbf{v}}- {\textbf{f}}_\varepsilon \cdot {\textbf{v}}\end{aligned}$$

where we use the abbreviated notation ${\textbf{A}}_\varepsilon := {\textbf{A}}_{\phi _\varepsilon }$, and ${\textbf{f}}_\varepsilon := {\textbf{f}}_{\phi _\varepsilon }$. Moreover, for $\varepsilon \ge 0$, we define the perturbed state ${\textbf{u}}_\varepsilon$ as the solution to

$$\begin{aligned} 0 = \partial _{\textbf{v}}L(\varepsilon , {\textbf{u}}_\varepsilon , {\textbf{v}}), \end{aligned}$$

i.e. ${\textbf{u}}_\varepsilon$ is the solution to

54 $$\begin{aligned} {\textbf{A}}_\varepsilon {\textbf{u}}_\varepsilon = {\textbf{f}}_\varepsilon \end{aligned}$$

and the (unperturbed) adjoint state ${\textbf{p}}$ as the solution to

55 $$\begin{aligned} 0 = \partial _{\textbf{u}}L(0, {\textbf{u}}, {\textbf{p}}), \end{aligned}$$

for the state ${\textbf{u}}$ given, i.e. ${\textbf{p}}$ solves

$$\begin{aligned} {\textbf{A}}^\top {\textbf{p}}= - \partial _{\textbf{u}}J(\phi , {\textbf{u}}). \end{aligned}$$

Note that we use the notation ${\textbf{u}}$ for ${\textbf{u}}_{\varepsilon = 0}$. The numerical topological-shape derivative at the node ${{\textbf{x}}}_k$ can be computed as the limit

$$\begin{aligned} d {\mathcal {J}}(\phi )({{\textbf{x}}}_k) = \underset{\varepsilon \searrow 0}{\text{ lim } } \frac{1}{|\varOmega (\phi _\varepsilon ) \triangle \varOmega (\phi )|} (J(\phi _\varepsilon , {\textbf{u}}_\varepsilon ) - J(\phi , {\textbf{u}}) ). \end{aligned}$$

With the help of the Lagrangian (53), we can rewrite the right hand side as

$$\begin{aligned} J(\phi _\varepsilon , {\textbf{u}}_\varepsilon ) - J(\phi ,{\textbf{u}}) = L(\varepsilon , {\textbf{u}}_\varepsilon , {\textbf{p}}) - L(0, {{\textbf{u}}}, {\textbf{p}}) \end{aligned}$$

where we used that ${\textbf{u}}_\varepsilon$ solves (54) for $\varepsilon \ge 0$. Following the approach used in [18], we use the fundamental theorem of calculus as well as (55) to rewrite this as

56 $$\begin{aligned} J(\phi _\varepsilon , {\textbf{u}}_\varepsilon ) - J(\phi ,{{\textbf{u}}}) =&\int _0^1 [\partial _{\textbf{u}}L(\varepsilon , {{\textbf{u}}}+ s({\textbf{u}}_\varepsilon - {{\textbf{u}}}), {\textbf{p}}) - \partial _{\textbf{u}}L(\varepsilon , {\textbf{u}}, {\textbf{p}})]({\textbf{u}}_\varepsilon - {{\textbf{u}}}) \text{ d }s \end{aligned}$$

57 $$\begin{aligned}&+[ \partial _{\textbf{u}}L(\varepsilon , {\textbf{u}}, {\textbf{p}}) - \partial _{\textbf{u}}L(0, {\textbf{u}}, {\textbf{p}})]({\textbf{u}}_\varepsilon - {\textbf{u}}) \end{aligned}$$

58 $$\begin{aligned}&+ L(\varepsilon , {\textbf{u}}, {\textbf{p}}) - L(0, {\textbf{u}}, {\textbf{p}}). \end{aligned}$$

Thus the numerical topological-shape derivative can be obtained as the sum of three limits,

$$\begin{aligned} d {\mathcal {J}}(\phi )({{\textbf{x}}}_k) = R_1({\textbf{u}}, {\textbf{p}}) + R_2({\textbf{u}}, {\textbf{p}}) + R_0({\textbf{u}}, {\textbf{p}}) \end{aligned}$$

where

$$\begin{aligned}&R_1({\textbf{u}}, {\textbf{p}}):= \underset{\varepsilon \searrow 0}{\text{ lim } } \frac{1}{|\varOmega (\phi _\varepsilon ) \triangle \varOmega (\phi )|} \int _0^1 [\partial _{\textbf{u}}L(\varepsilon , {{\textbf{u}}}+ s({\textbf{u}}_\varepsilon - {{\textbf{u}}}), {\textbf{p}}) - \partial _{\textbf{u}}L(\varepsilon , {\textbf{u}}, {\textbf{p}})]({\textbf{u}}_\varepsilon - {{\textbf{u}}}) \text{ d }s, \\&R_2({\textbf{u}}, {\textbf{p}}) := \underset{\varepsilon \searrow 0}{\text{ lim } } \frac{1}{|\varOmega (\phi _\varepsilon ) \triangle \varOmega (\phi )|}[ \partial _{\textbf{u}}L(\varepsilon , {\textbf{u}}, {\textbf{p}}) - \partial _{\textbf{u}}L(0, {\textbf{u}}, {\textbf{p}})]({\textbf{u}}_\varepsilon - {\textbf{u}}), \\&R_0({\textbf{u}}, {\textbf{p}}) := \underset{\varepsilon \searrow 0}{\text{ lim } } \frac{1}{|\varOmega (\phi _\varepsilon ) \triangle \varOmega (\phi )|} [ L(\varepsilon , {\textbf{u}}, {\textbf{p}}) - L(0, {\textbf{u}}, {\textbf{p}})]. \end{aligned}$$

For $J(\phi _\varepsilon , {\textbf{u}}) = c_2 ({\textbf{u}}- \hat{\textbf{u}}) {\tilde{{\textbf{M}}}}_\varepsilon ({\textbf{u}}- {\hat{{\textbf{u}}}})$, where ${\tilde{{\textbf{M}}}}_\varepsilon := {\tilde{{\textbf{M}}}}_{\phi _\varepsilon }$ represents the matrix ${\tilde{{\textbf{M}}}}$ defined in (24) with $\varOmega = \varOmega (\phi _\varepsilon )$, we get

$$\begin{aligned} R_1({\textbf{u}}, {\textbf{p}}) =&c_2 \underset{\varepsilon \searrow 0}{\text{ lim } } \frac{1}{|\varOmega (\phi _\varepsilon ) \triangle \varOmega (\phi )|} ({\textbf{u}}_\varepsilon - {{\textbf{u}}})^\top {\tilde{{\textbf{M}}}}_\varepsilon ({\textbf{u}}_\varepsilon - {{\textbf{u}}}). \end{aligned}$$

Moreover,

59 $$\begin{aligned} R_2({\textbf{u}}, {\textbf{p}}) =&\underset{\varepsilon \searrow 0}{\text{ lim } } \frac{1}{|\varOmega (\phi _\varepsilon ) \triangle \varOmega (\phi )|} \left[ 2 c_2 ({\tilde{{\textbf{M}}}}_\varepsilon - {\tilde{{\textbf{M}}}}) ({\textbf{u}}- {\hat{{\textbf{u}}}}) \cdot ({\textbf{u}}_\varepsilon - {{\textbf{u}}}) + ({\textbf{A}}_\varepsilon - {\textbf{A}}) ({\textbf{u}}_\varepsilon - {{\textbf{u}}}) \cdot {\textbf{p}}\right] , \end{aligned}$$

and

60 $$\begin{aligned} R_0({\textbf{u}}, {\textbf{p}}) =&\underset{\varepsilon \searrow 0}{\text{ lim } } \frac{1}{|\varOmega (\phi _\varepsilon ) \triangle \varOmega (\phi )|} \left[ c_2({{\textbf{u}}}- {\hat{{\textbf{u}}}})^\top ({\tilde{{\textbf{M}}}}_\varepsilon - {\tilde{{\textbf{M}}}}) ({{\textbf{u}}}- {\hat{{\textbf{u}}}}) + {\textbf{p}}^\top ({\textbf{A}}_\varepsilon - {\textbf{A}}) {\textbf{u}}- ({\textbf{f}}_\varepsilon - {\textbf{f}})\cdot {\textbf{p}}\right] . \end{aligned}$$

#### Lemma 2

There exist constants $c>0, \hat{\varepsilon }>0$ such that for all $\varepsilon \in (0,{\hat{\varepsilon }})$

$$\begin{aligned} \Vert {\textbf{u}}_\varepsilon - {{\textbf{u}}}\Vert \le c \, \varepsilon ^o. \end{aligned}$$

Here, $o=1$ in the case of a shape perturbation and $o=2$ in the case of a topological perturbation.

#### Proof

Subtracting (54) for $\varepsilon = 0$ from the same equation with $\varepsilon >0$, we get

$$\begin{aligned} {\textbf{A}}_\varepsilon ({\textbf{u}}_\varepsilon - {\textbf{u}})&= {\textbf{f}}_\varepsilon - {\textbf{f}}- ({\textbf{A}}_\varepsilon - {\textbf{A}}) {\textbf{u}}\end{aligned}$$

and thus, by the ellipticity of the bilinear form corresponding to ${\textbf{A}}_\varepsilon$ and the triangle inequality, there is a constant $c>0$ such that for all $\varepsilon >0$ small enough

61 $$\begin{aligned} \Vert {\textbf{u}}_\varepsilon - {\textbf{u}}\Vert \le c ( \Vert {\textbf{f}}_\varepsilon - {\textbf{f}}\Vert - \Vert {\textbf{A}}_\varepsilon - {\textbf{A}}\Vert \Vert {\textbf{u}}\Vert ). \end{aligned}$$

For the difference between the perturbed and unperturbed right hand sides we have

$$\begin{aligned} |({\textbf{f}}_\varepsilon - {\textbf{f}})_i| \le&|(f_1 - f_2)| \int _{\varOmega (\phi _\varepsilon ) \triangle \varOmega (\phi )} |\varphi _i(x)| \; \text{ d }x \\ |({\textbf{A}}_\varepsilon - {\textbf{A}})_{i,j}| \le&\int _{\varOmega (\phi _\varepsilon ) \triangle \varOmega (\phi )} \bigg ( |(\lambda _1 - \lambda _2)| |\nabla \varphi _j(x)| |\nabla \varphi _i(x)| + |(\alpha _1 - \alpha _2)| |\varphi _j(x)| |\varphi _i(x)| \bigg )\; \text{ d }x. \end{aligned}$$

The result follows from the boundedness of $|\varphi _i(x)|$ and $|\nabla \varphi _i(x)|$ together with (49) which implies the existence of $c>0$ such that $|\varOmega (\phi _\varepsilon ) \triangle \varOmega (\phi )| \le c \varepsilon ^o$ (cf. Remark 4). $\square$

From Lemma 2 it follows that the terms $R_1({\textbf{u}}, {\textbf{p}})$ and $R_2({\textbf{u}}, {\textbf{p}})$ vanish. We remark that this is in contrast to the continuous case, where asymptotic analysis shows that $R_2$ does not vanish. We will address this issue in more detail in Sect. 5. Thus, in the discrete setting we obtain $d {\mathcal {J}}(\phi )({{\textbf{x}}}_k)=R_0({\textbf{u}}, {\textbf{p}})$, i.e.,

62 $$\begin{aligned} d {\mathcal {J}}(\phi )({{\textbf{x}}}_k)&= \frac{\textbf{p}^\top (d_k {\textbf{A}} \,{{\textbf{u}}}- d_k {\textbf{f}}) +c_2 ({{\textbf{u}}}- \hat{\textbf{u}})^\top d_k{\tilde{{\textbf{M}}}} ({{\textbf{u}}}- {\hat{{\textbf{u}}}}) }{ d_k{\tilde{a}}} \end{aligned}$$

where

63 $$\begin{aligned} \begin{aligned} d_k {\textbf{A}}&= \lim _{\varepsilon \searrow 0} \frac{{\textbf{A}}_\varepsilon - {\textbf{A}}}{\varepsilon ^o},{} & {} {}&d_k {\tilde{{\textbf{M}}}}&= \lim _{\varepsilon \searrow 0} \frac{{\tilde{{\textbf{M}}}}_\varepsilon - {\tilde{{\textbf{M}}}}}{\varepsilon ^o}, \\ d_k {\textbf{f}}&= \lim _{\varepsilon \searrow 0} \frac{{\textbf{f}}_\varepsilon -{\textbf{f}}}{\varepsilon ^o},{} & {} {}&d_k {\tilde{a}}&= \lim _{\varepsilon \searrow 0} \frac{|\varOmega (\phi _\varepsilon )\triangle \varOmega (\phi )|}{\varepsilon ^o}, \end{aligned} \end{aligned}$$

with $o=1$ for ${{\textbf{x}}}_k\in {\mathfrak {S}}$ and $o =2$ for ${{\textbf{x}}}_k\in {\mathfrak {T}}^-\cup {\mathfrak {T}}^+$. To obtain (62), we divided both numerator and denominator of (60) by $\varepsilon ^o$ and used that the limit of the quotient coincides with the quotient of the limits provided both limits exist and the limit in the denominator does not vanish. Next we state the numerical topological-shape derivative of problem (2) for arbitrary constant weights $c_1, c_2 \ge 0$ in the cost function (1).

#### Theorem 2

(Numerical topological-shape derivative) For $l=1,\dots ,N$, let ${\textbf{u}}_l = [u_{l_1},u_{l_2},u_{l_3}]^\top$ and ${\textbf{p}}_l = [p_{l_1},p_{l_2},p_{l_3}]^\top$ be the nodal values for element $\tau _{l}$ of the solution and the adjoint, and

$$\begin{aligned} {\textbf{k}}_{0,l}[i,j] = \left( J_l^{-1}\nabla _\xi \psi _j\right) ^\top \left( J_l^{-1}\nabla _\xi \psi _i\right) , \quad i,j =1,\dots ,3. \end{aligned}$$

Moreover, $u_k = u({{\textbf{x}}}_k)$, $p_k = p({{\textbf{x}}}_k)$, and ${\hat{u}}_k = {\hat{u}}({{\textbf{x}}}_k)$. For ${{\textbf{x}}}_k \in {\mathfrak {T}}^-$ the numerical topological derivative reads

64 $$\begin{aligned} d{\mathcal {J}}(\phi )({{\textbf{x}}}_k) = -c_1 -\Delta \lambda \frac{\sum _{l \in I_{{{\textbf{x}}}_k}} \frac{{\textbf{p}}_{l}^\top {\textbf{k}}_{0,l} {\textbf{u}}_{l}\left| {\text {det} }J_l\right| }{\phi _{l_2}\phi _{l_3}}}{\sum _{l \in I_{{{\textbf{x}}}_k}}\frac{\left| {\text {det} }J_l\right| }{\phi _{l_2}\phi _{l_3}} } -\Delta \alpha p_ku_k +\Delta fp_k - c_2\Delta {\tilde{\alpha }}(u_k-{\hat{u}}_k)^2, \end{aligned}$$

whereas for ${{\textbf{x}}}_k \in {\mathfrak {T}}^+$ we have

65 $$\begin{aligned} d{\mathcal {J}}(\phi )({{\textbf{x}}}_k) = c_1 + \Delta \lambda \frac{\sum _{l \in I_{{{\textbf{x}}}_k}} \frac{{\textbf{p}}_{l}^\top {\textbf{k}}_{0,l} {\textbf{u}}_{l}\left| {\text {det} }J_l\right| }{\phi _{l_2}\phi _{l_3}}}{\sum _{l \in I_{{{\textbf{x}}}_k}}\frac{\left| {\text {det} }J_l\right| }{\phi _{l_2}\phi _{l_3}} } +\Delta \alpha p_ku_k -\Delta f p_k + c_2\Delta {\tilde{\alpha }}(u_k-{\hat{u}}_k)^2, \end{aligned}$$

with

$$\begin{aligned} \Delta \alpha = \alpha _1-\alpha _2, \quad \Delta \lambda = \lambda _1-\lambda _2, \quad \Delta f = f_1-f_2, \quad \Delta {\tilde{\alpha }} = {\tilde{\alpha }}_1-{\tilde{\alpha }}_2 . \end{aligned}$$

For ${{\textbf{x}}}_k \in {\mathfrak {S}}$ the numerical shape derivative reads

66 $$\begin{aligned}&d{\mathcal {J}}(\phi )({{\textbf{x}}}_k) = -c_1 + \Delta \lambda \frac{\sum _{l \in C_k} {\textbf{p}}_{l}^\top {\textbf{k}}_{0,l} {\textbf{u}}_{l} \,d_k a_{l}}{d_k{\tilde{a}}} +\Delta \alpha \frac{\sum _{l \in C_k} {\textbf{p}}_{l}^\top d_k{\textbf{m}}^I_{l} \,\textbf{u}_{l}}{d_k{\tilde{a}}}\nonumber \\&\quad -\Delta f \frac{\sum _{l \in C_k} {\textbf{p}}_{l}^\top d_k\textbf{f}_l^I }{d_k{\tilde{a}}} +c_2\Delta {\tilde{\alpha }} \frac{\sum _{l \in C_k} ({\textbf{u}}_{l}-\hat{{\textbf{u}}}_{l})^\top d_k{\textbf{m}}^I_{l} \,({\textbf{u}}_{l}-\hat{{\textbf{u}}}_{l})}{d_k{\tilde{a}}}, \end{aligned}$$

where the entries of the element matrix $d_k{\textbf{m}}_{l}^I$ and of the element vector $d_k{\textbf{f}}_{l}^I$ are dependent on the local cut situation (cases $I=A^\pm$, $B^\pm$, $C^\pm$) and are given in Appendix A.2. The values $d_k {\tilde{a}}$ can be computed by (48) considering Remark 4.

#### Proof

We evaluate (62) for ${{\textbf{x}}}_k \in {\mathfrak {T}}^-$ and $O_{k,\varepsilon }= T^{-\rightarrow +}_{k,\varepsilon }$. Thus, $o=2$ in (63). We note that

67 $$\begin{aligned} {\textbf{p}}^\top d_k {\textbf{A}} \,{{\textbf{u}}}= \sum _{l=1}^N \textbf{p}_{l}^\top (d_k{\textbf{m}}_{l} +d_k{\textbf{k}}_{l}) {\textbf{u}}_{l}. \end{aligned}$$

We have for $l\in I_{{{\textbf{x}}}_k}$

$$\begin{aligned} {\textbf{k}}_{l}(\phi _{\varepsilon })-{\textbf{k}}_l(\phi )&= \textbf{k}_{0,l} \left| {\text {det} }J_l\right| \bigg ( \int _{\xi _1=0}^1\int _{\xi _2=0}^{1-\xi _1} \lambda _1 H(-\phi _{\varepsilon }\circ \Phi _l) +\lambda _2 H(\phi _{\varepsilon }\circ \Phi _l) \;\textrm{d} \xi _2 \;\textrm{d} \xi _1 \\&\quad -\int _{\xi _1=0}^1\int _{\xi _2=0}^{1-\xi _1} \lambda _1 H(-\phi \circ \Phi _l) +\lambda _2 H(\phi \circ \Phi _l) \;\textrm{d} \xi _2 \;\textrm{d} \xi _1\bigg ) \\&={\textbf{k}}_{0,l} \left| {\text {det} }J_l\right| \bigg ( \lambda _1\int _{\xi _1=0}^1\int _{\xi _2=0}^{1-\xi _1} (H(-\phi _{\varepsilon }\circ \Phi _l) -H(-\phi \circ \Phi _l)) \;\textrm{d} \xi _2 \;\textrm{d} \xi _1 \\&\quad +\lambda _2\int _{\xi _1=0}^1\int _{\xi _2=0}^{1-\xi _1} (H(\phi _\varepsilon \circ \Phi _l) - H(\phi \circ \Phi _l)) \;\textrm{d} \xi _2 \;\textrm{d} \xi _1\bigg )\\&= {\textbf{k}}_{0,l} (\lambda _1 - \lambda _2)\delta _{k,\varepsilon } a_l \end{aligned}$$

due to (31) because $|\varOmega _\varepsilon |-|\varOmega | = -(|D {\setminus } \varOmega _\varepsilon |-|D {\setminus } \varOmega |)$ and with (38) we obtain

68 $$\begin{aligned} d_k{\textbf{k}}_l = \underset{\varepsilon \searrow 0}{\text{ lim } } \frac{{\textbf{k}}_l(\phi _\varepsilon ) - {\textbf{k}}_l(\phi )}{\varepsilon ^2}&= -\Delta \lambda \frac{\left| {\text {det} }J_l\right| }{2\phi _{l_2}\phi _{l_3}} \textbf{k}_{0,l}. \end{aligned}$$

Due to

$$\begin{aligned} \int _{\xi _1=0}^{l_1} \int _{\xi _2=0}^{l_2\left( 1-\frac{\xi _1}{l_1}\right) } \xi _1^a \xi _2^b \;\textrm{d} \xi _2 \;\textrm{d} \xi _1 = \frac{\varepsilon ^{a+b+2}}{\phi _{l_2}^{a+1}\phi _{l_3}^{b+1} + O(\varepsilon ^{a+b+2})} \end{aligned}$$

for some $a,b \in {\mathbb {N}}$, we have

69 $$\begin{aligned} \begin{aligned} d_k{\textbf{m}}_l = \lim _{\varepsilon \searrow 0} \frac{\textbf{m}_{l}(\phi _{\varepsilon })-{\textbf{m}}_l(\phi )}{\varepsilon ^2}&= - \lim _{\varepsilon \searrow 0} \frac{\Delta \alpha }{\varepsilon ^2} \int _{\xi _1=0}^{l_1} \int _{\xi _2=0}^{l_2\left( 1-\frac{\xi _1}{l_1}\right) } \psi _i(\xi )\psi _j(\xi ) \left| {\text {det} }J_l\right| \;\textrm{d} \xi _2 \;\textrm{d} \xi _1 \\&= \begin{bmatrix} -\frac{\Delta \alpha \left| {\text {det} }J_l\right| }{2\phi _{l_2}\phi _{l_3}} &{} 0 &{} 0 \\ 0 &{} 0 &{} 0 \\ 0 &{} 0 &{} 0 \end{bmatrix}, \end{aligned} \end{aligned}$$

and conclude

70 $$\begin{aligned} \sum _{l=1}^N {\textbf{p}}_{l}^\top d_k{\textbf{m}}_{l} {\textbf{u}}_{l} = -\Delta \alpha p_k u_k\sum _{l \in I_{{{\textbf{x}}}_k}} \frac{\left| {\text {det} }J_l\right| }{2\phi _{l_2}\phi _{l_3}}. \end{aligned}$$

Furthermore, with

71 $$\begin{aligned} \begin{aligned} d_k{\textbf{f}}_l =\lim _{\varepsilon \searrow 0} \frac{\textbf{f}_{l}(\phi _{\varepsilon })-{\textbf{f}}_l(\phi )}{\varepsilon ^2}&= - \lim _{\varepsilon \searrow 0} \frac{\Delta f}{\varepsilon ^2} \int _{\xi _1=0}^{l_1} \int _{\xi _2=0}^{l_2\left( 1-\frac{\xi _1}{l_1}\right) } \psi _i(\xi ) \left| {\text {det} }J_l\right| \;\textrm{d} \xi _2 \;\textrm{d} \xi _1 \\&= \begin{bmatrix} -\frac{\Delta f\left| {\text {det} }J_l\right| }{2\phi _{l_2}\phi _{l_3}} \\ 0 \\ 0 \end{bmatrix}, \end{aligned} \end{aligned}$$

it follows that

72 $$\begin{aligned} {\textbf{p}}^\top d_k{\textbf{f}} = \sum _{l=1}^N {\textbf{p}}_{l}^\top d_k{\textbf{f}}_{l} = -\Delta f p_k \sum _{l \in I_{{{\textbf{x}}}_k}} \frac{\left| {\text {det} }J_l\right| }{2\phi _{l_2}\phi _{l_3}}. \end{aligned}$$

Analogously to (69) we have

73 $$\begin{aligned} \begin{aligned} d_k\tilde{{\textbf{m}}}_l = \lim _{\varepsilon \searrow 0} \frac{\tilde{{\textbf{m}}}_{l}(\phi _{\varepsilon })-\tilde{\textbf{m}}_l(\phi )}{\varepsilon ^2} = \begin{bmatrix} -\frac{\Delta {\tilde{\alpha }}\left| {\text {det} }J_l\right| }{2\phi _{l_2}\phi _{l_3}} &{} 0 &{} 0 \\ 0 &{} 0 &{} 0 \\ 0 &{} 0 &{} 0 \end{bmatrix}, \end{aligned} \end{aligned}$$

and obtain

74 $$\begin{aligned} \begin{aligned} ({{\textbf{u}}}- {\hat{{\textbf{u}}}})^\top d_k{\tilde{{\textbf{M}}}} ({{\textbf{u}}}- {\hat{{\textbf{u}}}})&= \sum _{l \in I_{{{\textbf{x}}}_k}} ({\textbf{u}}_{0,l} - {\hat{{\textbf{u}}}}_l)^\top d_k\tilde{\textbf{m}}_l ({\textbf{u}}_{0,l} - {\hat{{\textbf{u}}}}_l) \\ {}&= -\Delta {\tilde{\alpha }}(u_k-{\hat{u}}_k)^2 \sum _{l \in I_{{{\textbf{x}}}_k}} \frac{\left| {\text {det} }J_l\right| }{2\phi _{l_2}\phi _{l_3}}. \end{aligned} \end{aligned}$$

In the present situation, $d_k{\tilde{a}}$ is given by the absolute value of (38) (see also Remark 4),

75 $$\begin{aligned} d_k{\tilde{a}} = \sum _{l \in I_{{{\textbf{x}}}_k}^{A_+}}\frac{\left| {\text {det} }J_l\right| }{2\phi _{l_2}\phi _{l_3}}. \end{aligned}$$

By inserting (68), (70), (72), (74), and (75) in (62), together with Corollary 2, we obtain the sought expression (64). Formula (65) can be obtained in an analogous way as (64).

The formula in (66) follows directly from (62) together with Corollary 2. The values of $d_k {\textbf{m}}^I_l$ and $d_k \textbf{f}^I_l$ for all possible cut situations $I \in \{A^+, A^-, B^+, B^-, C^+, C^-\}$ are given in Appendix A.2 and were computed using symbolic computer algebra tools. A mathematical derivation of these terms is omitted here for brevity. $\square$

#### Remark 5

In this work, we restricted ourselves to the spatially two-dimensional setting $D \subset {\mathbb {R}}^2$ where *D* is discretized by a triangular mesh. We expect that, at the cost of more technical computations, an extension to three-dimensional problems involving a tetrahedral mesh can be carried out along the lines of the presented work. For shape perturbations, one has to consider all different configurations how a tetrahedral element can be divided by a planar interface. In addition to the class of configurations where one of the four nodes is cut off (cf. Fig. 3 for the 2d case), one also has to consider all configurations where any two of the four vertices of the tetrahedron are on one side of the interface and two on the other side. For topological perturbations, only the first class of configurations will be of interest and no significant difference to the two-dimensional setting is expected.

Similarly, an extension of the presented method to more general settings such as more general cost functions of the type $g = g(\varOmega , u, \nabla u)$ or spatially varying material coefficients can be obtained at the cost of more technical computations.

## Connection Between Continuous and Discrete Sensitivities

The topological-shape derivative introduced in (25) and computed for model problem (2) in Theorem 2 represents a sensitivity of the discretized problem (23). In this section, we draw some comparisons with the classical topological and shape derivatives defined on the continuous level before discretization. While the purpose of this paper is to follow the idea *discretize-then-differentiate*, we consider the other way here for comparison reasons.

### Connections Between Continuous and Discrete Topological Derivative

For comparison, we also illustrate the derivation of the continuous topological derivative according to (4) for problem (2). We use the same Lagrangian framework as introduced in Sect. 4.2, see also [18]. Given a shape $\varOmega \in {\mathcal {A}}$, a point $z \in D {\setminus } \partial \varOmega$, an inclusion shape $\omega \subset {\mathbb {R}}^d$ with $0 \in \omega$ and $\varepsilon \ge 0$, we define the inclusion $\omega _\varepsilon = z + \varepsilon \omega$ and the perturbed Lagrangian

$$\begin{aligned}&G(\varepsilon , \varphi , \psi ) := c_1 |\varOmega _\varepsilon | + c_2 \int _D \tilde{\alpha }_{\varOmega _\varepsilon } | \varphi - {\hat{u}}|^2\; \text{ d }x \\&\quad + \int _D \lambda _{\varOmega _\varepsilon } \nabla \varphi \cdot \nabla \psi + \alpha _{\varOmega _\varepsilon } \varphi \psi \;\text{ d }x - \int _D f_{\varOmega _\varepsilon } \psi \; \text{ d }x \end{aligned}$$

where $\varOmega _\varepsilon = \varOmega {\setminus } \omega _\varepsilon$ for $z \in \varOmega$ and $\varOmega _\varepsilon = \varOmega \cup \omega _\varepsilon$ for $z \in D {\setminus } {\overline{\varOmega }}$. For simplicity, we only consider the latter case, *i.e.* $z \in D \setminus {\overline{\varOmega }}$ in the sequel.

Noting that $u_\varepsilon$, $\varepsilon \ge 0$, is the solution to the perturbed state equation with parameter $\varepsilon$, the topological derivative can also be written as

76 $$\begin{aligned} d_T{\mathfrak {g}}(\varOmega )(z) = \underset{\varepsilon \searrow 0}{\text{ lim } }\frac{1}{|\omega _\varepsilon |} (G(\varepsilon , u_\varepsilon , p) - G(0, u_0, p)) \end{aligned}$$

with the solution to the unperturbed adjoint state equation *p*. As in Sect. 4.2, this leads to the topological derivative consisting of the three terms

$$\begin{aligned} d_T{\mathfrak {g}}(\varOmega )(z) = R_1(u, p) + R_2(u, p) + R_0(u, p) \end{aligned}$$

where

$$\begin{aligned} R_1(u, p) :=&\underset{\varepsilon \searrow 0}{\text{ lim } } \frac{1}{|\varOmega _\varepsilon \triangle \varOmega |} \int _0^1 [\partial _u G(\varepsilon , u_0 + s(u_\varepsilon - u_0), p) - \partial _u G(\varepsilon , u, p)](u_\varepsilon - u_0) \text{ d }s, \\ R_2(u, p) :=&\underset{\varepsilon \searrow 0}{\text{ lim } } \frac{1}{|\varOmega _\varepsilon \triangle \varOmega |}[ \partial _u G(\varepsilon , u, p) - \partial _u G(0, u, p)](u_\varepsilon - u), \\ R_0(u, p) :=&\underset{\varepsilon \searrow 0}{\text{ lim } } \frac{1}{|\varOmega _\varepsilon \triangle \varOmega |} [ G(\varepsilon , u, p) - G(0, u, p)], \end{aligned}$$

provided that these limits exist. It is straightforwardly checked that for $z \in D \setminus {\overline{\varOmega }}$

$$\begin{aligned} R_0(u,p)&= c_1 + c_2 ({\tilde{\alpha }}_1 - {\tilde{\alpha }}_2) (u - {\hat{u}})^2(z) + (\lambda _1 - \lambda _2) \nabla u(z) \cdot \nabla p(z)\\&\quad + (\alpha _1 - \alpha _2) u(z) p(z) - (f_1-f_2)(z) p(z). \end{aligned}$$

For the term $R_2(u,p)$, we obtain

$$\begin{aligned} R_2(u,p) =&\underset{\varepsilon \searrow 0}{\text{ lim } } \frac{1}{|\omega _\varepsilon |} \bigg [ 2 c_2 \int _{\omega _\varepsilon } ({\tilde{\alpha }}_{1} - \tilde{\alpha }_2) (u_0 - {\hat{u}})(u_\varepsilon - u_0) \; dy\\&\quad + \int _{\omega _\varepsilon } (\lambda _{1} - \lambda _2) \nabla (u_\varepsilon - u_0) \cdot \nabla p \; dy \\&\quad + \int _{\omega _\varepsilon } (\alpha _{1} - \alpha _2) (u_\varepsilon - u_0) p \; dy \bigg ]. \end{aligned}$$

A change of variables $y = T_\varepsilon (x) = z + \varepsilon x$ yields

77 $$\begin{aligned} \begin{aligned} R_2(u,p)&= \underset{\varepsilon \searrow 0}{\text{ lim } } \frac{1}{|\omega |} \bigg [ 2 c_2 ({\tilde{\alpha }}_{1} - \tilde{\alpha }_2)\int _{\omega } (u_0 - {\hat{u}})\circ T_\varepsilon (u_\varepsilon - u_0) \circ T_\varepsilon \; dx \\ {}&\quad + (\lambda _{1} - \lambda _2) \int _{\omega } (\nabla (u_\varepsilon - u_0) )\circ T_\varepsilon \cdot (\nabla p)\circ T_\varepsilon \; dx \\&\quad + (\alpha _{1} - \alpha _2) \int _{\omega } (u_\varepsilon - u_0)\circ T_\varepsilon \, p\circ T_\varepsilon \; dx \bigg ] \end{aligned} \end{aligned}$$

We have a closer look at the diffusion term

78 $$\begin{aligned} R_2^\lambda (u,p):= \underset{\varepsilon \searrow 0}{\text{ lim } } \frac{1}{|\omega |} (\lambda _1 - \lambda _2)\int _\omega (\nabla (u_\varepsilon - u_0))\circ T_\varepsilon \cdot (\nabla p)\circ T_\varepsilon \; \text{ d }x. \end{aligned}$$

In the continuous setting, we now define $K_\varepsilon := \frac{1}{\varepsilon }(u_\varepsilon - u_0)\circ T_\varepsilon$ and use the chain rule $(\nabla \varphi )\circ T_\varepsilon = \frac{1}{\varepsilon }\nabla (\varphi \circ T_\varepsilon )$ to obtain

79 $$\begin{aligned} R_2^\lambda (u,p)=&\underset{\varepsilon \searrow 0}{\text{ lim } } \frac{1}{|\omega |} (\lambda _1 - \lambda _2) \int _\omega \nabla K_\varepsilon \cdot (\nabla p) \circ T_\varepsilon \; \text{ d }x \end{aligned}$$

Next, one can show the weak convergence $\nabla K_\varepsilon \rightharpoonup \nabla K$ for $K \in \dot{BL}({\mathbb {R}}^2)$ being defined as the solution to the exterior problem

$$\begin{aligned} \int _{{\mathbb {R}}^d} \lambda _\omega \nabla K \cdot \nabla \psi \; \text{ d }x = -(\lambda _1 - \lambda _2) \int _\omega \nabla u(z) \cdot \nabla \psi \; \text{ d }x \quad \text{ for } \text{ all } \psi \in \dot{BL}({\mathbb {R}}^2), \end{aligned}$$

where $\dot{BL}({\mathbb {R}}^2):= \{v \in H^1_{loc}({\mathbb {R}}^2): \nabla v \in L^2({\mathbb {R}}^2) \}/_{{\mathbb {R}}}$ is a Beppo-Levi space. Assuming continuity of $\nabla p$ around the point of perturbation *z*, it follows that

80 $$\begin{aligned} R_2^\lambda (u,p) = \frac{1}{|\omega |} (\lambda _1 - \lambda _2) \int _\omega \nabla K \cdot \nabla p(z)\; \text{ d }x. \end{aligned}$$

It can be shown that the other terms in (77) vanish and thus $R_2(u,p) = R_2^\lambda (u,p)$. Finally, it follows from the analysis in [18, Sec. 5] that $R_1(u,p) + R_2(u,p) = \frac{1}{|\omega |}(\lambda _1 - \lambda _2) \int _\omega \nabla K \cdot \nabla p(z) \, \text{ d }x = R_2(u,p)$, thus $R_1(u,p) = 0$ and $d_T{\mathfrak {g}}(\varOmega )(z) = R_0(u,p) + R_2^\lambda (u,p)$.

#### Remark 6

Comparing the topological derivative formula obtained here with the sensitivity for interior nodes ${{\textbf{x}}}_k \in {\mathfrak {T}}^-\cup {\mathfrak {T}}^+$ obtained in Sect. 4, we see that the term corresponding to $R_2^\lambda (u,p)$, i.e., the term

$$\begin{aligned} \underset{\varepsilon \searrow 0}{\text{ lim } } \frac{1}{|\varOmega (\phi _\varepsilon )\triangle \varOmega (\phi )|}({\textbf{K}}_\varepsilon - {\textbf{K}}_0) ({\textbf{u}}_\varepsilon - {\textbf{u}}_0) \cdot {\textbf{p}}\end{aligned}$$

in (59), vanishes in the discrete setting. This can be seen as follows: For $u_\varepsilon ^h$, $\varepsilon \ge 0$, and $p^h \in V_h$, we have the expansion in the finite element basis

$$\begin{aligned} u_\varepsilon ^h(x) = \sum _{i=1}^M u_\varepsilon ^{(i)} \varphi _i , \qquad p^h(x) = \sum _{i=1}^M p^{(i)} \varphi _i . \end{aligned}$$

If we now plug in these discretized functions into (78) and consider a fixed mesh size *h*, we get on the other hand

$$\begin{aligned}&R_2^\lambda (u^h, p^h) = \underset{\varepsilon \searrow 0}{\text{ lim } } \frac{1}{|\omega |} (\lambda _1 - \lambda _2) \int _\omega (\nabla (u_\varepsilon ^h - u_0^h))\circ T_\varepsilon \cdot (\nabla p^h)\circ T_\varepsilon \; {\text{ dx }} \\&\quad = \underset{\varepsilon \searrow 0}{\text{ lim } } \frac{1}{|\omega |}(\lambda _1 - \lambda _2) \sum _{i,j=1}^M (u_\varepsilon ^{(i)}-u_0^{(i)})p^{(j)} \int _\omega (\nabla \varphi _i)(z+\varepsilon x) \cdot (\nabla \varphi _j)(z+\varepsilon x) \; \text{ d }x = 0 \end{aligned}$$

where we used the continuity of $\varepsilon \mapsto {\textbf{u}}_\varepsilon$ according to Lemma 2. Note that, since the mesh and the basis functions are assumed to be fixed and independent of $\varepsilon$, unlike in the continuous setting, here the continuity of the normal flux of the discrete solution $u_\varepsilon ^h$ across the interface $\partial \omega _\varepsilon$ is not preserved. We mention that, when using an extended discretization technique that accounts for an accurate resolution of the material interface (e.g. XFEM [25] or CutFEM [11]), the corresponding discrete sensitivities would include a term corresponding to $R_2^\lambda (u,p)$.

### Connection Between Continuous and Discrete Shape Derivative

The continuous shape derivative $d_S{\mathfrak {g}}(\varOmega )(V)$ for a PDE-constrained shape optimization problem given a shape $\varOmega \in {\mathcal {A}}$ and a smooth vector field *V* can also be obtained via a Lagrangian approach. For our problem (2), it can be obtained as $d_S{\mathfrak {g}}(\varOmega )(V) = \partial _t G(0, u, p)$ with

$$\begin{aligned} G(t, \varphi , \psi ) :=&c_1 \int _\varOmega \xi (t) \; \text{ d }x + c_2 \int _D \tilde{\alpha }_{\varOmega } | \varphi - {\hat{u}}\circ T_t|^2 \xi (t) \; \text{ d }x \\&+ \int _D \lambda _{\varOmega } A(t) \nabla \varphi \cdot \nabla \psi + \alpha _{\varOmega } \varphi \psi \xi (t) \;\text{ d }x- \int _D f_{\varOmega } \psi \xi (t)\; \text{ d }x \end{aligned}$$

where $T_t(x) = x + t V(x)$, $\xi (t) = \text{ det }(\partial T_t)$, $A(t) = \xi (t) \partial T_t^{-1} \partial T_t^{-T}$, see [31] for a detailed description. In the continuous setting, the shape derivative reads in its volume form

$$\begin{aligned} d_S{\mathfrak {g}}(\varOmega )(V) = \int _D {\mathcal {S}}_1^{\varOmega } : \partial V + {\mathcal {S}}_0^\varOmega \cdot V \; \text{ d }x \end{aligned}$$

with ${\mathcal {S}}_1^\varOmega$ and ${\mathcal {S}}_0^\varOmega$ given in (11) and (12), respectively. Under certain smoothness assumptions, it can be transformed into the Hadamard or boundary form

81 $$\begin{aligned} d_S{\mathfrak {g}}(\varOmega )(V) = \int _{\partial \varOmega } L \, (V\cdot n) \; \text{ d }S_x \end{aligned}$$

with $L =( {\mathcal {S}}_1^{\varOmega , \text {in}} - {\mathcal {S}}_1^{\varOmega , \text {out}})n \cdot n$ given by

82 $$\begin{aligned} L = c_1 + c_2({\tilde{\alpha }}_1 - {\tilde{\alpha }}_2) (u - {\hat{u}})^2 + (\alpha _1 - \alpha _2) u p - (f_1 - f_2) p + L^\lambda \end{aligned}$$

where $L^\lambda$ is given by

83 $$\begin{aligned}&L^\lambda := (\lambda _1- \lambda _2) (\nabla u \cdot \tau )(\nabla p \cdot \tau ) - \left( \frac{1}{\lambda _1} - \frac{1}{\lambda _2}\right) ( \lambda _\varOmega \nabla u \cdot n)( \lambda _\varOmega \nabla p \cdot n) \nonumber \\&=\lambda _1 \nabla u_{\text {in}} \cdot \nabla p_{\text {in}} - \lambda _2 \nabla u_{\text {out}} \cdot \nabla p_{\text {out}} \nonumber \\&- 2 \lambda _1 (\nabla u_{\text {in}}\cdot n) ( \nabla p_{\text {in}} \cdot n) + 2 \lambda _2 (\nabla u_{\text {out}}\cdot n) ( \nabla p_{\text {out}} \cdot n). \end{aligned}$$

Here, $\nabla u_{\text {in}}, \nabla p_{\text {in}}$ and $\nabla u_{\text {out}}, \nabla p_{\text {out}}$ denote the restrictions of the gradients to $\varOmega$ and $D {\setminus } {\overline{\varOmega }}$, respectively, and *n* denotes the unit normal vector pointing out of $\varOmega$. Note that, when using a finite element discretization which does not resolve the interface such that the gradients of the discretized state and adjoint variable are continuous, i.e. $\nabla u_{h, \text {in}} = \nabla u_{h, \text {out}}$ and $\nabla p_{h, \text {in}} = \nabla p_{h, \text {out}}$, (83) becomes

84 $$\begin{aligned} L^\lambda _h = (\lambda _1 - \lambda _2) \nabla u_h \cdot \nabla p_h - 2(\lambda _1 - \lambda _2) (\nabla u_h \cdot n)(\nabla p_h \cdot n) \end{aligned}$$

We now discretize the continuous shape derivative formula (81) for the vector field $V^{(k)}$ that is obtained from the perturbation of the level set function $\phi$ in (only) node ${{\textbf{x}}}_k$, $k \in {\mathfrak {S}}$ fixed. For that purpose we fix $\phi \in S_h^1(D)$ and the corresponding domain $\varOmega (\phi )$. Note that we consider $V^{(k)}$ to be supported only on the discretized material interface $\partial \varOmega (\phi ) \cap D$. We begin with the case of the pure volume cost function by setting $c_2=0$.

#### Proposition 1

Let $c_2=0$ and ${{\textbf{x}}}_k \in {\mathfrak {S}}$ fixed. Let $V^{(k)}$ the vector field that corresponds to a perturbation of the value of $\phi$ at position ${{\textbf{x}}}_k$. Then

$$\begin{aligned} d_S{\mathfrak {g}}(\varOmega (\phi ))(V^{(k)}) = c_1 \sum _{l \in C_k} d_ka_l \end{aligned}$$

where $d_k a_l$ is as in (48).

#### Proof

For $c_2=0$ we also have $p=0$ and thus $L = c_1$, i.e., we are in the case of pure volume minimization. From (81) we know that $d_S{\mathfrak {g}}(\varOmega (\phi ))(V^{(k)}) = c_1 \int _{\partial \varOmega (\phi )} V^{(k)} \cdot n \; \text{ d }S_x$. First of all, we note that the vector field $V^{(k)}$ corresponding to a perturbation of $\phi$ at node $x_k$ is only nonzero in elements $\tau _l$ for $l \in C_k$ with $C_k$ as defined in (32). Thus, the shape derivative reduces to

$$\begin{aligned} d_S{\mathfrak {g}}(\varOmega (\phi ))(V^{(k)}) = c_1 \sum _{l \in C_k} \int _{\tau _l \cap \partial \varOmega (\phi )} V^{(k)}\cdot n \; \text{ d }S_x. \end{aligned}$$

We compute the vector field $V^{(k)}$ and normal vector *n* explicitly depending on the cut situation. Recall the sets $I_{{{\textbf{x}}}_k}^{A_+}, I_{{{\textbf{x}}}_k}^{A_-}, I_{{{\textbf{x}}}_k}^{B_+}, I_{{{\textbf{x}}}_k}^{B_-}, I_{{{\textbf{x}}}_k}^{C_+}, I_{{{\textbf{x}}}_k}^{C_-}$ introduced in (34), see also Fig. 3.

Given two points ${\textbf{p}}$ and ${\textbf{q}}$ and their respective level set values *a* and *b* of different sign, $ab<0$, we denote the root of the linear interpolating function by

$$\begin{aligned} {\textbf{y}}({\textbf{p}}, {\textbf{q}}, a, b) = {\textbf{p}}- \frac{a}{b-a}({\textbf{q}}- {\textbf{p}}) \end{aligned}$$

and note that $\frac{d}{da} {\textbf{y}}({\textbf{p}}, {\textbf{q}}, a, b) = - \frac{b}{(b-a)^2}({\textbf{q}}- {\textbf{p}})$.

We begin with Configuration A. For an element index $l \in I_{{{\textbf{x}}}_k}^{A_+}\cup I_{{{\textbf{x}}}_k}^{A_-}$, we denote the vertices of element $\tau _l$ by ${{\textbf{x}}}_{l_1}, {{\textbf{x}}}_{l_2}, {{\textbf{x}}}_{l_3}$ and assume their enumeration in counter-clockwise order with ${{\textbf{x}}}_k = {{\textbf{x}}}_{l_1}$. The corresponding values of the given level set function $\phi$ are denoted by $\phi _{l_1}, \phi _{l_2}, \phi _{l_3}$, respectively. We parametrize the line connecting the two roots of the perturbed level set function along the edges by

$$\begin{aligned} p^A[\varepsilon ](s) = (1-s) {\textbf{y}}({{\textbf{x}}}_{l_1}, {{\textbf{x}}}_{l_2}, \phi _{l_1}+\varepsilon , \phi _{l_2}) + s {\textbf{y}}({{\textbf{x}}}_{l_1}, {{\textbf{x}}}_{l_3}, \phi _{l_1}+\varepsilon ,\phi _{l_3}) \end{aligned}$$

and obtain the vector field corresponding to the perturbation of $\phi _{k} = \phi _{l_1}$ along the line $\tau _l \cap \partial \varOmega (\phi )$ as

$$\begin{aligned} {\hat{V}}^A(s) = \frac{d}{d\varepsilon } p^A[\varepsilon ](s)|_{\varepsilon = 0} = (1-s) \frac{- \phi _{l_2}}{(\phi _{l_2}-\phi _{l_1})^2} ({{\textbf{x}}}_{l_2} - {{\textbf{x}}}_{l_1}) + s \frac{- \phi _{l_3}}{ (\phi _{l_3} - \phi _{l_1})^2} ({{\textbf{x}}}_{l_3}-{{\textbf{x}}}_{l_1}). \end{aligned}$$

Introducing the notation $d_{ki,kj}:= |{\textbf{y}}({{\textbf{x}}}_{l_k}, {{\textbf{x}}}_{l_i}, \phi _{l_k}, \phi _{l_i})-{\textbf{y}}({{\textbf{x}}}_{l_k}, {{\textbf{x}}}_{l_j}, \phi _{l_k}, \phi _{l_j}) |$ for the length of the interface in element $\tau _l$, the normed tangential vector along $\tau _l \cap \partial \varOmega (\phi )$ and the normed normal vector pointing out of $\varOmega (\phi )$ are given by

$$\begin{aligned} {\hat{t}}^A =&\frac{{\textbf{y}}({{\textbf{x}}}_{l_1}, {{\textbf{x}}}_{l_3}, \phi _{l_1}, \phi _{l_3}) - {\textbf{y}}({{\textbf{x}}}_{l_1}, {{\textbf{x}}}_{l_2}, \phi _{l_1}, \phi _{l_2}) }{d_{13,12}} \\ {\hat{n}}^{A_+} =&\frac{\phi _{l_1}}{d_{13,12}} \left( \frac{1}{\phi _{l_2} - \phi _{l_1}} R ({{\textbf{x}}}_{l_2} - {{\textbf{x}}}_{l_1})- \frac{1}{\phi _{l_3} - \phi _{l_1}} R ({{\textbf{x}}}_{l_3} - {{\textbf{x}}}_{l_1}) \right) =-{\hat{n}}^{A_-} \end{aligned}$$

where *R* denotes a 90 degree counter-clockwise rotation matrix, $$R = \begin{pmatrix} 0&{} -1\\ 1 &{} 0 \end{pmatrix}$$. Noting that $({{\textbf{x}}}_{l_3} - {{\textbf{x}}}_{l_1})^\top R ({{\textbf{x}}}_{l_2} - {{\textbf{x}}}_{l_1}) = |\text{ det }J_l| = - ({{\textbf{x}}}_{l_2} - {{\textbf{x}}}_{l_1})^\top R ({{\textbf{x}}}_{l_3} - {{\textbf{x}}}_{l_1})$ and $({{\textbf{x}}}_{l_j} - {{\textbf{x}}}_{l_1})^\top R ({{\textbf{x}}}_{l_j} - {{\textbf{x}}}_{l_1}) = 0$, $j=2,3$, we get

85 $$\begin{aligned} {\hat{V}}^A(s) \cdot {\hat{n}}^{A_+} = -\frac{|\text{ det } J_l| \phi _{l_1}}{d_{13,12}} \frac{\phi _{l_2}(\phi _{l_3}-\phi _{l_1}) + s \phi _{l_1}(\phi _{l_2} - \phi _{l_3})}{(\phi _{l_2}-\phi _{l_1})^2(\phi _{l_3}-\phi _{l_1})^2} = - {\hat{V}}^A(s) \cdot {\hat{n}}^{A_-} . \end{aligned}$$

Finally, by elementary computation we obtain for $l \in I_{{{\textbf{x}}}_k}^{A_+}$

$$\begin{aligned} \int _{\tau _l \cap \partial \varOmega (\phi )} V^{(k)} \cdot n \; \text{ d }S_x =&d_{13,12} \int _0^1 {\hat{V}}^A(t) \cdot {\hat{n}}^{A_+} \; \text{ d }t \\ =&| \text{ det } J_l| \phi _{l_1} \frac{-\phi _{l_2}\phi _{l_3} + \frac{1}{2} \phi _{l_1}(\phi _{l_2}+ \phi _{l_3})}{(\phi _{l_2}-\phi _{l_1})^2(\phi _{l_3}-\phi _{l_1})^2} \end{aligned}$$

and the same formula with a different sign for $l \in I_{{{\textbf{x}}}_k}^{A_-}$. Proceeding analogously, we obtain for $l \in I_{{{\textbf{x}}}_k}^{B_+}$ and $l \in I_{{{\textbf{x}}}_k}^{C_+}$

86 $$\begin{aligned} {\hat{V}}^B(s) \cdot {\hat{n}}^{B_+} =&\frac{|\text{ det } J_l|}{d_{23,21}} \frac{(1-s) \phi _{l_2}^2 }{(\phi _{l_2} - \phi _{l_1})^2 (\phi _{l_3} - \phi _{l_2})} = - {\hat{V}}^B(s) \cdot {\hat{n}}^{B_-}, \end{aligned}$$

87 $$\begin{aligned} {\hat{V}}^C(s) \cdot {\hat{n}}^{C_+} =&\frac{|\text{ det } J_l|}{d_{31,32}} \frac{(1-s) \phi _{l_3}^2}{(\phi _{l_3}-\phi _{l_1})^2 (\phi _{l_2}-\phi _{l_3})} = -{\hat{V}}^C(s) \cdot {\hat{n}}^{C_-}, \end{aligned}$$

and further

$$\begin{aligned} \int _{\tau _l \cap \partial \varOmega (\phi )} V^{(k)} \cdot n \; \text{ d }S_x=&\frac{|\text{ det }J_l|}{2} \frac{\phi _{l_2}^2}{(\phi _{l_2}-\phi _{l_1})^2(\phi _{l_3}-\phi _{l_2})}, \quad l \in I_{{{\textbf{x}}}_k}^{B_+}, \\ \int _{\tau _l \cap \partial \varOmega (\phi )} V^{(k)} \cdot n \; \text{ d }S_x =&\frac{|\text{ det }J_l|}{2} \frac{-\phi _{l_3}^2}{(\phi _{l_3}-\phi _{l_1})^2(\phi _{l_3}-\phi _{l_2})}, \quad l \in I_{{{\textbf{x}}}_k}^{C_+}, \end{aligned}$$

respectively. Again, the formulas for $l \in I_{{{\textbf{x}}}_k}^{B_-}$ and $l \in I_{{{\textbf{x}}}_k}^{C_-}$ just differ by a different sign.

Finally, comparing the computed values with the formulas of $d_k a_l$ (48) yields the claimed result. $\square$

In view of Proposition 1, the definition in (13) and Remark 4, we see that, in the case $c_2=0$, it holds

88 $$\begin{aligned} \hat{d}_S{\mathfrak {g}}(\varOmega (\phi ))(V^{(k)}) = \frac{c_1 \sum _{l\in C_k} d_ka_l}{\sum _{l\in C_k} d_k {\tilde{a}}_l} = - c_1, \end{aligned}$$

which is in alignment with the first term of the formula in (66).

Next, we consider the general PDE-constrained case where $c_2>0$.

#### Proposition 2

Let $c_1 = 0$ and ${{\textbf{x}}}_k \in {\mathfrak {S}}$ fixed. Let $V^{(k)}$ the vector field that corresponds to a perturbation of the value of $\phi$ at position ${{\textbf{x}}}_k$. Then

89 $$\begin{aligned} \begin{aligned} d_S{\mathfrak {g}}(\varOmega (\phi ))(V^{(k)}) =&\; \; \;\; \, \Delta \lambda \sum _{l \in C_k} {\textbf{p}}_{l}^\top {\textbf{k}}_{0,l} \textbf{u}_{l} \,d_k a_{l} + \Delta \alpha \sum _{l \in C_k} \textbf{p}_{l}^\top d_k{\textbf{m}}^I_{l} \,{\textbf{u}}_{l}\\&- \Delta f \sum _{l \in C_k} {\textbf{p}}_{l}^\top d_k{\textbf{f}}_l^I +c_2 \Delta {\tilde{\alpha }} \sum _{l \in C_k} (\textbf{u}_{l}-\hat{{\textbf{u}}}_{l})^\top d_k{\textbf{m}}^I_{l} \,(\textbf{u}_{l}-\hat{{\textbf{u}}}_{l}) \\&-2 \Delta \lambda \sum _{l \in C_k}(\nabla u_h \cdot n^I)|_{\tau _l \cap \partial \varOmega (\phi )}(\nabla p_h \cdot n)|_{\tau _l \cap \partial \varOmega (\phi )} d_ka_l, \end{aligned} \end{aligned}$$

where we use the same notation as in Theorem 2. In particular, $d_k {\textbf{m}}^I_l$ and $d_k {\textbf{f}}^I_l$ depend on the cut situation, $I \in \{A^+, A^-, B^+, B^-, C^+, C^-\}$ and are given explicitly in Appendix A.2.

#### Proof

Let an element index $l \in C_k$ fixed and ${\textbf{u}}_l = [u_{l_1}, u_{l_2}, u_{l_3}]^\top$, ${\textbf{p}}_l = [p_{l_1}, p_{l_2}, p_{l_3}]^\top$ contain the nodal values of the finite element functions $u_h$ and $p_h$ corresponding to the three vertices ${{\textbf{x}}}_{l_1}$, ${{\textbf{x}}}_{l_2}$, ${{\textbf{x}}}_{l_3}$ of element *l*, respectively. Also here, the ordering is in counter-clockwise direction starting with ${{\textbf{x}}}_{l_1} = {{\textbf{x}}}_k$. We compute the shape derivative (81) with *L* given in (82) after discretization (i.e. after replacing the functions *u*, *p*, ${\hat{u}}$ by finite element approximations $u_h$, $p_h$, ${\hat{u}}_h$). In particular, the term $L^\lambda$ is approximated by (84). Depending on how the material interface $\partial \varOmega (\phi )$ cuts through element $\tau _l$, the normal component of the vector field $V^{(k)}$ along the line $\tau _l \cap \partial \varOmega (\phi )$ is given in (85)–(87). For and $I \in \{A^+, A^-, B^+, B^-, C^+, C^-\}$, it can be seen by elementary yet tedious calculations that

$$\begin{aligned} \int _{\tau _l \cap \partial \varOmega (\phi )} p_h(x) V^{I}(x) \cdot n^{I} \, \text{ d }S_x =&\; {\textbf{p}}_l^\top d_k {\textbf{f}}_l^I,\\ \int _{\tau _l \cap \partial \varOmega (\phi )} u_h(x) p_h(x) V^{I}(x) \cdot n^{I} \, \text{ d }S_x =&\; {\textbf{p}}_l^\top d_k {\textbf{m}}^I_l {\textbf{u}}_l,\\ \int _{\tau _l \cap \partial \varOmega (\phi )} (u_h(x)-{\hat{u}}_h(x))^2 V^{I}(x) \cdot n^{I} \, \text{ d }S_x =&\; ({\textbf{u}}_l - \hat{\textbf{u}}_l)^\top d_k {\textbf{m}}^I_l ({\textbf{u}}_l - \hat{{\textbf{u}}}_l), \end{aligned}$$

with $d_k {\textbf{m}}^I_l$ and $d_k {\textbf{f}}^I_l$ as given in Appendix A.2. Examplarily, we illustrate the calculation for the second of these terms for the cut situation $I = A^+$, see Fig. 3a. Let $u_{l,12}$ and $u_{l,13}$ denote the values of the linear function $u_h|_{\tau _l}$ at the intersection of the interface $\partial \varOmega (\phi )$ with the edges that connect the point ${{\textbf{x}}}_{l_1}$ with ${{\textbf{x}}}_{l_2}$ and ${{\textbf{x}}}_{l_1}$ with ${{\textbf{x}}}_{l_3}$, respectively. Note the relations $u_{l,12} = \frac{u_{l_2} \phi _{l_1} - u_{l_1} \phi _{l_2}}{\phi _{l_1}-\phi _{l_2}}$ and $u_{l,13} = \frac{u_{l_3} \phi _{l_1} - u_{l_1} \phi _{l_3}}{\phi _{l_1}-\phi _{l_3}}$. Analogously we define the values $p_{l,12}$ and $p_{l,13}$. The function $u_h$ along the line $\tau _l \cap \partial \varOmega (\phi )$ can now be written as ${\hat{u}}_h(s) = u_{l,12} + s (u_{l,13} - u_{l,12})$, $s \in [0,1]$ and we get

$$\begin{aligned} \int _{\tau _l \cap \partial \varOmega (\phi )}&u_h(x) p_h(x) V^{I}(x) \cdot n^{I} \, \text{ d }S_x \\&= d_{13,12} \int _0^1 (u_{l,12} + s (u_{l,13} - u_{l,12})) (p_{l,12} + s (p_{l,13} - p_{l,12})) {\hat{V}}^{A^+}(s) \cdot {\hat{n}}^{A^+} \; \text{ d }s \\&= {\textbf{p}}_l^\top d_k {\textbf{m}}^{A^+}_l {\textbf{u}}_l \end{aligned}$$

where $d_{13,12} = |\tau _l \cap \partial \varOmega (\phi )|$. The last equality is obtained by plugging in (85) and straightforward (yet tedious) calculation. Finally, since $u_h$ and $p_h$ are linear and the normal vector is constant on $\tau _l \cap \partial \varOmega (\phi )$, we see that $L_h^\lambda$ is constant and, using Proposition 1, we obtain

$$\begin{aligned}&\int _{\tau _l \cap \partial \varOmega (\phi )} L_h^\lambda (x) V^I(x)\cdot n^I \; \text{ d }S_x = L_h^\lambda (x) \int _{\tau _l \cap \partial \varOmega (\phi )} V^I(x)\cdot n^I \; \text{ d }S_x \\&\quad =\Delta \lambda \left( {\textbf{p}}_l^\top {\textbf{k}}_{0,l} \textbf{u}_l -2 (\nabla u_h \cdot n^I)|_{\tau _l \cap \partial \varOmega (\phi )}(\nabla p_h \cdot n^I)|_{\tau _l \cap \partial \varOmega (\phi )} \right) d_ka_l. \end{aligned}$$

$$\square $$

Combining the findings of Propositions 1 and 2 and dividing by $d_k {\tilde{a}}$ (defined in Remark 4), we obtain the resulting formula for the alternative definition of the shape derivative as defined in (13)

90 $$\begin{aligned}{} & {} \hat{d}_S{\mathfrak {g}}(\varOmega (\phi ))(V^{(k)}) = -c_1 + \frac{1}{d_k {\tilde{a}}} \Bigg ( \Delta \lambda \sum _{l \in C_k} {\textbf{p}}_{l}^\top {\textbf{k}}_{0,l} {\textbf{u}}_{l} \,d_k a_{l} \nonumber \\{} & {} \quad + \Delta \alpha \sum _{l \in C_k} {\textbf{p}}_{l}^\top d_k\textbf{m}^I_{l} \,{\textbf{u}}_{l} - \Delta f \sum _{l \in C_k} \textbf{p}_{l}^\top d_k{\textbf{f}}_l^I \nonumber \\{} & {} \quad +c_2 \Delta {\tilde{\alpha }} \sum _{l \in C_k} (\textbf{u}_{l}-\hat{{\textbf{u}}}_{l})^\top d_k{\textbf{m}}^I_{l} \,(\textbf{u}_{l}-\hat{{\textbf{u}}}_{l}) \nonumber \\{} & {} -2 \Delta \lambda \sum _{l \in C_k}(\nabla u_h \cdot n^I)|_{\tau _l \cap \partial \varOmega (\phi )}(\nabla p_h \cdot n)|_{\tau _l \cap \partial \varOmega (\phi )} d_ka_l \Bigg ). \end{aligned}$$

#### Remark 7

Note that (90) is obtained by discretizing the continuous shape derivative (81)–(83). We see immediately that (90) resembles the formula for the discrete topological-shape derivative for nodes ${{\textbf{x}}}_k \in {\mathfrak {S}}$ (66). The only difference is the occurence of the last term in (90), which is not accounted for when performing the sensitivity analysis in the discrete setting.

Note that this term stems from the presence of $\partial T_t^{-1} \partial T_t^{-T}$ in the matrix *A*(*t*), which, in turn, originates from two applications of the chain rule, $(\nabla \varphi ) \circ T_t = \partial T_t^{-\top } \nabla (\varphi \circ T_t)$. Similarly as in the case of the topological derivative in Sect. 5.1, the reason for this discrepancy is the fact that, for the given discretization scheme, the gradients of the finite element basis functions are constant on each element and thus $(\nabla \varphi ) \circ T_t = \nabla \varphi$ for small enough shape perturbation parameter *t*.

#### Remark 8

As a second, conceptual difference between the classical continuous shape derivative defined by (8) and our discrete counterpart (66), we recall that in the definition (25) we divided by the change of volume also in the case ${{\textbf{x}}}_k \in {\mathfrak {S}}$.

Thus, comparing (90) with (66), we see that, for the chosen finite element setting, the discretization of the continuous shape derivative coincides with the discrete topological-shape derivative for the volume function, but not for the PDE-constrained part of the cost function (1) when the diffusion term is perturbed.

## Numerical Experiments

In this section, we verify our implementation of the numerical topological-shape derivative derived in Sect. 4 by numerical experiments, before applying a level-set based topology optimization algorithm based on these sensitivities to our model problem.

### Verification

The implementation of the topological-shape derivative is verified by comparing the computed sensitivity values against numerical values obtained by three different approaches. These are (i) a finite difference test, (ii) an application of the complex step derivative [24] and (iii) a test based on hyper-dual numbers developed in [16]. All tests are conducted for a fixed configuration.

We recall the definition of the topological-shape derivative (25) at a node ${{\textbf{x}}}_k$ of the mesh

91 $$\begin{aligned} d{\mathcal {J}}(\phi )({{\textbf{x}}}_k) = \lim _{\varepsilon \searrow 0} \delta {\mathcal {J}}_{\varepsilon }(\phi )({{\textbf{x}}}_k) \quad \text{ with } \quad \delta {\mathcal {J}}_{\varepsilon }(\phi )({{\textbf{x}}}_k) := \frac{{\mathcal {J}}({O_{k,\varepsilon }} \phi )-{\mathcal {J}}(\phi )}{|\varOmega ({O_{k,\varepsilon }}\phi ) \triangle \varOmega (\phi )|} \end{aligned}$$

where $O_{k,\varepsilon }$ represents the operator $T^{-\rightarrow +}_{k,\varepsilon }$, $T^{+\rightarrow -}_{k,\varepsilon }$, $S_{k,\varepsilon }$ depending on whether the node ${{\textbf{x}}}_k$ is in ${\mathfrak {T}}^-$, ${\mathfrak {T}}^+$ or ${\mathfrak {S}}$, respectively.

#### Finite Difference Test

**Fig. 5:**
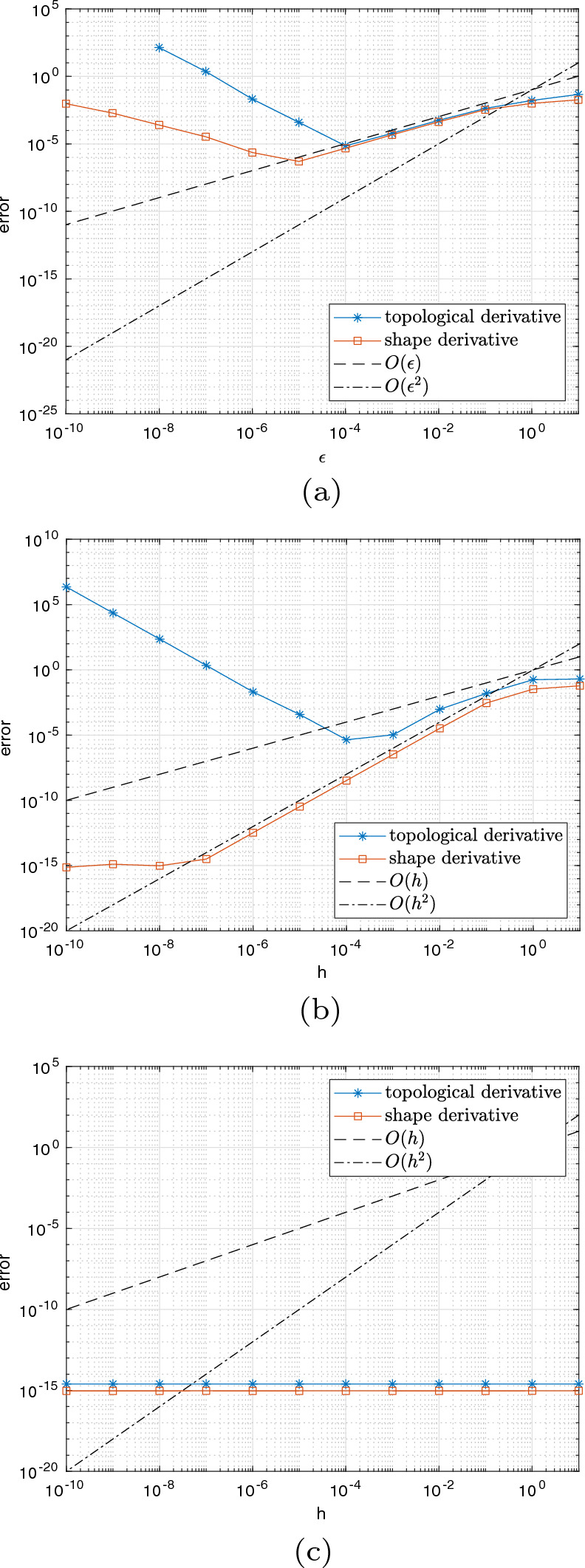
**a** Results of the finite difference test. **b** Results obtained with the complex step derivative. **c** Results obtained with hyper-dual numbers

For the finite difference (FD) test, we compute the errors

92 $$\begin{aligned} e_S^{FD}(\varepsilon )&= \sqrt{\sum _{{{\textbf{x}}}_k \in {\mathfrak {S}}} (\delta {\mathcal {J}}_{\varepsilon }(\phi )({{\textbf{x}}}_k) - d{\mathcal {J}}(\phi )({{\textbf{x}}}_k))^2}, \nonumber \\ e_T^{FD}(\varepsilon )&= \sqrt{\sum _{{{\textbf{x}}}_k \in {\mathfrak {T}}^-\cup {\mathfrak {T}}^+} (\delta {\mathcal {J}}_{\varepsilon }(\phi )({{\textbf{x}}}_k) - d{\mathcal {J}}(\phi )({{\textbf{x}}}_k))^2} \end{aligned}$$

for a decreasing sequence of values for $\varepsilon$. The results are shown in Fig. 5a. We observe convergence of order $\varepsilon$ up to a point where the cancellation error dominates.

#### Complex Step Derivative Test

In order to overcome this drawback of the finite difference test, we next consider a test based on the complex step (CS) derivative [24]. For the case of classical first order derivatives, this method is not subject to subtractive cancellation errors. For that purpose, using Remark 4, let us first rewrite (91) as

93 $$\begin{aligned} d{\mathcal {J}}(\phi )({{\textbf{x}}}_k) = \frac{\lim _{\varepsilon \searrow 0}\frac{{\mathcal {J}}({O_{k,\varepsilon }} \phi )-{\mathcal {J}}(\phi )}{\varepsilon ^o}}{\lim _{\varepsilon \searrow 0}\frac{|\varOmega ({O_{k,\varepsilon }}\phi ) \triangle \varOmega (\phi )|}{\varepsilon ^o}} =\frac{1}{d_k {\tilde{a}}} \lim _{\varepsilon \searrow 0}\frac{{\mathcal {J}}({O_{k,\varepsilon }} \phi )-{\mathcal {J}}(\phi )}{\varepsilon ^o}, \end{aligned}$$

where $o=1$ if ${{\textbf{x}}}_k \in {\mathfrak {S}}$ and $o=2$ if ${{\textbf{x}}}_k \in {\mathfrak {T}}^-\cup {\mathfrak {T}}^+$. Moreover, assuming a higher order expansion of the form

94 $$\begin{aligned} {\mathcal {J}}(O_{k,\varepsilon }\phi )&= {\mathcal {J}}(\phi ) + \varepsilon ^o \, d_k {\tilde{a}} \, d {\mathcal {J}}(\phi )({{\textbf{x}}}_k) \nonumber \\&\quad + \varepsilon ^{o+1} \, d_k {\tilde{a}} \, d^2 {\mathcal {J}}(\phi )({{\textbf{x}}}_k) + \varepsilon ^{o+2} \, d_k {\tilde{a}} \, d^3 {\mathcal {J}}(\phi )({{\textbf{x}}}_k) + {\mathfrak {o}}(\varepsilon ^{o+2}) \end{aligned}$$

with some higher order sensitivities $d^2 {\mathcal {J}}(\phi )({{\textbf{x}}}_k)$, $d^3 {\mathcal {J}}(\phi )({{\textbf{x}}}_k)$ and assuming that (94) also holds for complex-valued $\varepsilon$, we can follow the idea of the complex step derivative [24]: Setting $\varepsilon = ih$ in (94) with $h>0$ and *i* the complex unit yields

95 $$\begin{aligned} d {\mathcal {J}}(\phi )({{\textbf{x}}}_k) = \frac{\text {Im}({\mathcal {J}}(O_{k,ih}\phi ))}{h \, d_k {\tilde{a}}} + {\mathcal {O}}(h^2) \end{aligned}$$

in the case $o=1$ where ${{\textbf{x}}}_k \in {\mathfrak {S}}$, and

$$\begin{aligned} d {\mathcal {J}}(\phi )({{\textbf{x}}}_k) = \frac{\text {Re}({\mathcal {J}}(O_{k,ih}\phi )-{\mathcal {J}}(\phi ))}{-h^2 \, d_k {\tilde{a}}} + {\mathcal {O}}(h^2) \end{aligned}$$

in the case $o=2$ where ${{\textbf{x}}}_k \in {\mathfrak {T}}^-\cup {\mathfrak {T}}^+$. This means

$$\begin{aligned} d {\mathcal {J}}(\phi )({{\textbf{x}}}_k) = \delta {\mathcal {J}}_h^{CS}(\phi )({{\textbf{x}}}_k) + {\mathcal {O}}(h^2) \end{aligned}$$

with

96 $$\begin{aligned} \delta {\mathcal {J}}_h^{CS}(\phi )({{\textbf{x}}}_k) := {\left\{ \begin{array}{ll} \frac{\text {Re}({\mathcal {J}}(T^{-\rightarrow +}_{k, ih}\phi )-{\mathcal {J}}(\phi ))}{-h^2 \, d_k \tilde{a}}, &{} {{\textbf{x}}}_k \in {\mathfrak {T}}^-,\\ \frac{\text {Re}({\mathcal {J}}(T^{+\rightarrow -}_{k,ih}\phi )-{\mathcal {J}}(\phi ))}{-h^2 \, d_k \tilde{a}}, &{} {{\textbf{x}}}_k \in {\mathfrak {T}}^+,\\ \frac{\text {Im}({\mathcal {J}}( S_{k,ih}\phi ))}{h \, d_k {\tilde{a}}}, &{} {{\textbf{x}}}_k \in {\mathfrak {S}}. \end{array}\right. } \end{aligned}$$

Analogously to (92), we define the summed errors $e_S^{CS}(h)$ and $e_T^{CS}(h)$ by just replacing $\delta {\mathcal {J}}_\varepsilon (\phi )({{\textbf{x}}}_k)$ by $\delta {\mathcal {J}}_h^{CS}(\phi )({{\textbf{x}}}_k)$ defined above. Figure 5b shows the errors $e_S^{CS}$ and $e_T^{CS}$ for a positive, decreasing sequence of *h* where we observe quadratic decay for both errors. While the error $e_S^{CS}$ corresponding to the shape nodes ${{\textbf{x}}}_k \in {\mathfrak {S}}$ decays to machine precision, the error $e_T^{CS}$ corresponding to the interior nodes ${{\textbf{x}}}_k \in {\mathfrak {T}}^-\cup {\mathfrak {T}}^+$ deteriorates at some point due to the cancellation error occurring when subtracting ${\mathcal {J}}(\phi )$ from ${\mathcal {J}}(O_{k,ih}\phi )$ in (96).

##### Remark 9

In this subsection, for the sake of code verification, we have assumed a complex-valued perturbation of the level-set function. This has to be understood in a fully numerical context, rather than in the continuos setting. In particular, the representation of the domain by the level-set function (18) is not applicable any more, due to the occurrence of complex numbers in a comparison of numbers. However, within the numerical method to solve the state equation and to evaluate the objective function, comparison of numbers is only needed for the classification of ’cut elements’. Apart from classification the finite element method (computing local matrices, assembly of the global matrices and solving the system of linear equations) uses only formulas consisting of basic operations ($+$,−,$\cdot$, : ), where complex values are not a problem. For instance, the area of an element that is cut by a perturbed level-set function is written as an analytic formula depending on the perturbation parameter $\varepsilon$ in (35). Here, a complex valued perturbation parameter can be treated without problems.

When it comes to element classification the code has to follow the same execution path for the complex perturbation as if the perturbation were only real valued. To this end we distinguish between nodes belonging to ${\mathfrak {S}}$ and nodes belonging to ${\mathfrak {T}}^-\cup {\mathfrak {T}}^+$. In the case of a node ${{\textbf{x}}}_k\in {\mathfrak {S}}$ simply the real part of the complex level-set value can be used. This is due to the fact that for small enough perturbations the cut situation is the same as for the unperturbed setting. In the case of nodes ${{\textbf{x}}}_k$ belonging to ${\mathfrak {T}}^-$ and ${\mathfrak {T}}^+$, the operators $T^{+\rightarrow -}_{k,ih}$ and $T^{-\rightarrow +}_{k, ih}$ provide numbers with vanishing real part at ${{\textbf{x}}}_k$, *i.e.* $\text {Re}(T^{+\rightarrow -}_{k,ih}\phi ({{\textbf{x}}}_k))=\text {Re}(T^{-\rightarrow +}_{k, ih}\phi ({{\textbf{x}}}_k))=0$. Here, the imaginary part of the nodal value of the perturbed level-set function has the same sign as in the case of a purely real perturbation and can thus be used in the classification.

#### Test Based on Hyper-Dual Numbers

In order to overcome this cancellation error also for the case of ${{\textbf{x}}}_k \in {\mathfrak {T}}^-\cup {\mathfrak {T}}^+$, we resort to hyper-dual (HD) numbers as introduced in [16]. Here, the idea is to consider numbers with three non-real components denoted by $E_1$, $E_2$ and $E_1 E_2$ with $E_1^2 = E_2^2 = (E_1E_2)^2=0$. Assuming that expansion (94) holds up to order $o+1$ also for such hyper-dual values of $\varepsilon$, we can set $\varepsilon = h E_1 + h E_2 + 0 E_1E_2$ for some $h>0$. For $o=1$, considering only the first non-real part (i.e., the $E_1$part) and exploiting that $E_1^2 = 0$, we obtain the equality

97 $$\begin{aligned} d {\mathcal {J}}(\phi )({{\textbf{x}}}_k) = \frac{E_1\text {part}({\mathcal {J}}(O_{k, h E_1 + h E_2}\phi ))}{h \, d_k {\tilde{a}}} \end{aligned}$$

for ${{\textbf{x}}}_k \in {\mathfrak {S}}$. Similarly, with the same choice of $\varepsilon$, by considering only the $E_1E_2$-part of the expansion and exploiting $E_1^2=E_2^2=E_1^2E_2^2=0$, we obtain for $o=2$

98 $$\begin{aligned} d {\mathcal {J}}(\phi )({{\textbf{x}}}_k) = \frac{E_1E_2\text {part}({\mathcal {J}}(O_{k, h E_1 + h E_2}\phi ))}{2 h^2 \, d_k {\tilde{a}}} \end{aligned}$$

for ${{\textbf{x}}}_k \in {\mathfrak {T}}^-\cup {\mathfrak {T}}^+$. In this case, the corresponding summed errors $e_S^{HD}(h)$ and $e_T^{HD}(h)$ vanish for arbitrary $h \in {\mathbb {R}}$. This is also observed numerically since both (97) and (98) suffer neither from a truncation nor a cancellation error. Figure 5c shows that the the obtained results agree up to machine precision with the derivatives obtained by (64), (65), and the respective formula for the shape derivative (66).

##### Remark 10

Analogously to Remark 9, the perturbation of the level-set function by a hyper-dual number has to be understood in the numerical setting. We have implemented the hyper-dual numbers using operator overloading, *i.e.* fundamental operations were extended to work also on hyper-dual numbers respecting the calculation rules described in [16]. Thus formulas involving basic operations do not pose a problem. Nevertheless, care has to be taken when it comes to element classification. Again, in the case of nodes belonging to ${\mathfrak {S}}$ simply the real part of the hyper-dual number can be used. For topological perturbations the classification is based on the $E_1$part.

### Application of Optimization Algorithm to Model Problem

Finally we show the use of the numerical topological-shape derivative computed in Sect. 4 within a level-set based topology optimization algorithm. We first state the precise model problem, before introducing the algorithm and showing numerical results.

#### Problem Setting

We consider the unit square $D=[0,1]^2$ and minimize the objective function (1) with $c_1=0$ and $c_2=1$ subject to the PDE constraint in (2). The chosen problem parameters are shown in Table 1.

**Table 1:** Problem parameters for the numerical experiment

${\tilde{\alpha }}_1$	${\tilde{\alpha }}_2$	$\alpha _1$	$\alpha _2$	$\lambda _1$	$\lambda _2$	$f_1$	$f_2$
1	0.9	1	0.2	1	0.6	1	0.5

We consider a mixed Dirichlet-Neumann problem by choosing

$$\begin{aligned} \Gamma _D = \{(x,y)\in \partial D| y=0 \text{ or } y=1\}, \qquad \Gamma _N = \partial D \setminus \Gamma _D, \end{aligned}$$

and $$g_D(x,y) = y$$, $$g_N(x,y) = 0$$.

In order to define a desired state ${\hat{u}}$, we choose a level-set function $\phi _d$ which implies a desired shape $\varOmega _d$, compute the corresponding solution $u^*$ to $\phi _d$ and set ${\hat{u}}:= u^*$. Then, by construction, $(\varOmega _d, {\hat{u}})$ is also the solution of the design optimization problem. In the numerical tests we used five different meshes with 145, 545, 2113, 8321, and 33025 nodes respectively. For each mesh we obtain $\phi _d$ by interpolation of

99 $$\begin{aligned} {\bar{\phi }}_d(x,y) = \left( (x-0.3)^2+(y-0.4)^2-0.2^2\right) \left( (x-0.7)^2+(y-0.7)^2-0.1^2\right) . \nonumber \\ \end{aligned}$$

**Fig. 6:**
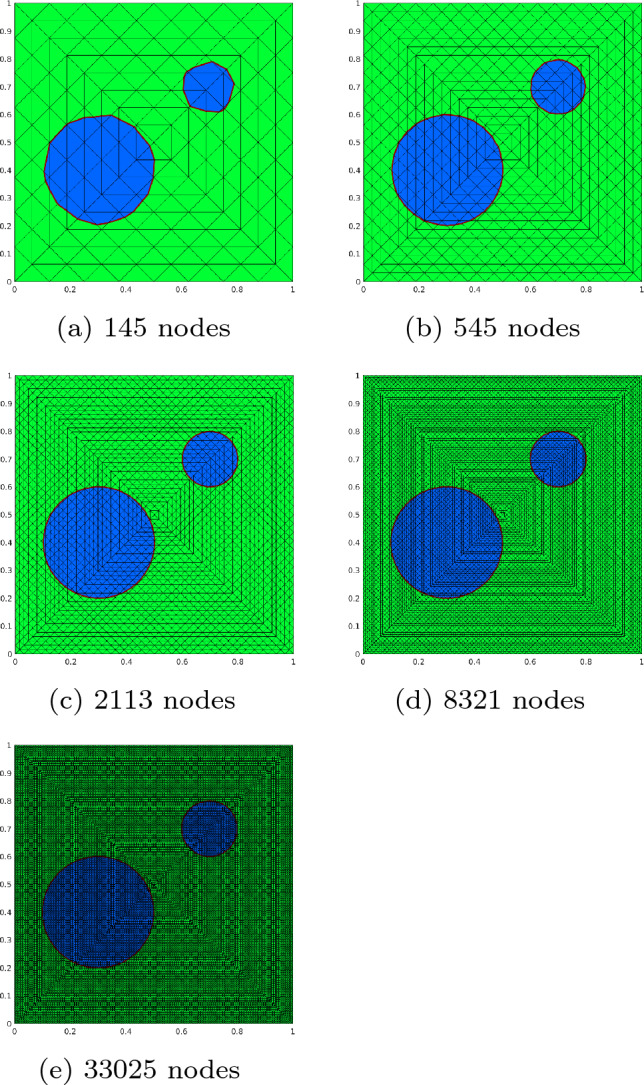
The different meshes and corresponding sought shapes used in the numerical experiments

This yields that $\varOmega$ are two (approximated) circles with radii 0.2 and 0.1 respectively, see Fig. 6.

#### Optimization Algorithm

The optimization algorithm we use to solve the problem introduced in Sect. 6.2.1 is inspired by [4].

##### Definition 1

We say a level set function $\phi \in S_h^1(D)$ is locally optimal for the problem described by ${\mathcal {J}}$ if

100 $$\begin{aligned} {\left\{ \begin{array}{ll} d {\mathcal {J}}(\phi )({{\textbf{x}}}_k) \ge 0 &{} \text {for } {{\textbf{x}}}_k \in {\mathfrak {T}}^-\cup {\mathfrak {T}}^+,\\ d {\mathcal {J}}(\phi )({{\textbf{x}}}_k) = 0 &{} \text {for } {{\textbf{x}}}_k \in {\mathfrak {S}}. \end{array}\right. } \end{aligned}$$

We introduce the generalized numerical topological-shape derivative $G_\phi \in S_h^1(D)$ with

101 $$\begin{aligned} G_\phi ({{\textbf{x}}}_k) = {\left\{ \begin{array}{ll} -\min (d{\mathcal {J}}(\phi )({{\textbf{x}}}_k),0) \quad &{}\text {for } {{\textbf{x}}}_k \in {\mathfrak {T}}^-, \\[8pt] \min (d{\mathcal {J}}(\phi )({{\textbf{x}}}_k),0) \quad &{}\text {for } {{\textbf{x}}}_k \in {\mathfrak {T}}^+, \\[8pt] -d{\mathcal {J}}(\phi )({{\textbf{x}}}_k) \quad &{}\text {for } {{\textbf{x}}}_k \in {\mathfrak {S}}. \end{array}\right. } \end{aligned}$$

With this definition, we immediately get the following optimality condition:

##### Lemma 3

Let $$\phi \in S_h^1(D)$$ and

102 $$\begin{aligned} G_\phi ({{\textbf{x}}}_k) = 0, \quad \text {for } k = 1,\dots ,M. \end{aligned}$$

Then $\phi$ is locally optimal in the sense of Definition 1.

The update of the level-set function based on the information of the topological-shape derivative is done by spherical linear interpolation (see also [4])

103 $$\begin{aligned} \phi _{i+1} = \frac{1}{\sin (\theta _i)}\left( \sin ((1-\kappa _i)\theta _i) \phi _i+ \sin (\kappa _i\theta _i)\frac{G_{\phi _i}}{\Vert G_{\phi _i}\Vert _{L_{2}(D)}} \right) , \end{aligned}$$

where $\theta _i = \text{ arc } \text{ cos }(( \phi _i, G_{\phi _i})_{L^2(D)})$ is the angle between the given level set function $\phi _i$ and the sensitivity $G_{\phi _i}$ in an $L^2(D)$-sense. Here, $\kappa _i\in (0,1)$ is a line search parameter which is adapted such that a decrease in the objective function is achieved. Note that, by construction, the update (103) preserves the $L^2(D)$-norm, $\Vert \phi _{i+1}\Vert _{L^2(D)} = \Vert \phi _{i}\Vert _{L^2(D)}$. As in [4, 17], we can also show that $\phi$ is evolving along a descent direction:

##### Lemma 4

Let $\phi _i, \phi _{i+1} \in S_h^1(D)$ two subsequent iterates related by (103). Then we have for ${{\textbf{x}}}_k \in {\mathfrak {T}}^-(\phi _i) \cup {\mathfrak {T}}^+(\phi _i)$

104 $$\begin{aligned} \phi _{i}({{\textbf{x}}}_k)> 0 > \phi _{i+1}({{\textbf{x}}}_k) \Longrightarrow d {\mathcal {J}}(\phi _i)({{\textbf{x}}}_k) <0, \end{aligned}$$

105 $$\begin{aligned} \phi _{i}({{\textbf{x}}}_k)< 0< \phi _{i+1}({{\textbf{x}}}_k) \Longrightarrow d {\mathcal {J}}(\phi _i)({{\textbf{x}}}_k) <0. \end{aligned}$$

##### Proof

Let ${{\textbf{x}}}_k \in {\mathfrak {T}}^+(\phi _i)$, i.e. $\phi _i({{\textbf{x}}}_k) > 0$ and assume that $\phi _{i+1}({{\textbf{x}}}_k) < 0$. Since $\text{ sin }(\theta )>0$ and $\text{ sin }(s\theta )>0$ for all $\theta \in (0, \pi )$ and $s\in (0,1)$, it follows from (103) that $G_{\phi _i}(x_k) < 0$ and thus, by (101), $d{\mathcal {J}}(\phi _i)({{\textbf{x}}}_k) < 0$ as claimed in (104). An analogous argument yields (105). $\square$

We can also show that $G_\phi$ constitutes a descent direction for ${{\textbf{x}}}_k \in {\mathfrak {S}}$.

##### Lemma 5

Let $\phi \in S^1_h(D)$ and suppose that

106 $$\begin{aligned} \underset{\varepsilon \searrow 0}{\text{ lim } } \frac{{\mathcal {J}}(S_{k,\varepsilon } \phi )-{\mathcal {J}}(\phi )}{|\varOmega (S_{k,\varepsilon } \phi ) \triangle \varOmega (\phi )|} = - \underset{\varepsilon \nearrow 0}{\text{ lim } } \frac{{\mathcal {J}}(S_{k,\varepsilon } \phi )-\mathcal J(\phi )}{|\varOmega (S_{k,\varepsilon } \phi ) \triangle \varOmega (\phi )|}. \end{aligned}$$

Let ${{\textbf{x}}}_k \in {\mathfrak {S}}(\phi )$ be fixed and let $\phi ^{\kappa }$ be the level set function according to (103) with line search parameter $\kappa \in (0,1)$ that is updated only in ${{\textbf{x}}}_k$, i.e., $\phi ^{\kappa } = a(\kappa ) \phi + b(\kappa ) G_{\phi } \varphi _k$ with $a(\kappa ) = \sin ((1-\kappa )\theta ) / \sin (\theta )$ and $b(\kappa ) = \sin (\kappa \theta ) / \left( \sin (\theta ) \Vert G_{\phi }\Vert _{L_{2}(D)} \right)$. Moreover assume that $|d \mathcal J(\phi )({{\textbf{x}}}_k)|>0$. Then there exists ${\overline{\kappa }} \in (0,1)$ such that for all $\kappa \in (0,{\overline{\kappa }})$

$$\begin{aligned} {\mathcal {J}}(\phi ^\kappa ) < {\mathcal {J}}(\phi ). \end{aligned}$$

##### Proof

Suppose that $0 > d {\mathcal {J}}(\phi )({{\textbf{x}}}_k)$. Then it follows from (25) that ${\mathcal {J}}(\phi + \varepsilon \varphi _k) < {\mathcal {J}}(\phi )$ for $\varepsilon >0$ small enough. Thus, since $a(\kappa ), b(\kappa )>0$ for $\theta \in (0, \pi )$ and $\kappa \in (0,1)$ and since ${\mathcal {J}}(\phi ^\kappa ) = \mathcal J(\frac{1}{a(\kappa )} \phi ^\kappa )$, it follows

$$\begin{aligned} {\mathcal {J}}(\phi ^\kappa ) = {\mathcal {J}}(\phi + b(\kappa )/a(\kappa ) G_{\phi } \varphi _k) = {\mathcal {J}}(\phi _i - b(\kappa )/a(\kappa ) d {\mathcal {J}}(\phi _i)({{\textbf{x}}}_k) \varphi _k) < {\mathcal {J}}(\phi _i) \end{aligned}$$

for $\kappa >0$ small enough since $-b(\kappa )/a(\kappa ) d\mathcal J(\phi _i)({{\textbf{x}}}_k)>0$ and $b(\kappa )/a(\kappa ) \rightarrow 0$ as $\kappa \searrow 0$. On the other hand, if $0 < d \mathcal J(\phi _i)({{\textbf{x}}}_k)$, it follows from (25) and (106) that ${\mathcal {J}}(\phi + \varepsilon \varphi _k) < {\mathcal {J}}(\phi )$ for $\varepsilon <0$ small enough and further for $\kappa$ small enough

$$\begin{aligned} {\mathcal {J}}(\phi ^\kappa ) = {\mathcal {J}}(\phi - b(\kappa )/a(\kappa ) d {\mathcal {J}}(\phi )({{\textbf{x}}}_k) \varphi _k) < {\mathcal {J}}(\phi ). \end{aligned}$$

$$\square $$

##### Remark 11

In the continuous setting, the property corresponding to (106) is fulfilled for smooth domains which can be seen as follows. Let $\varOmega _t^V = (\text {id}+t V)(\varOmega )$ and note that $\varOmega _{-t}^V = \varOmega _t^{-V}$. Then, by Lemma 1,

$$\begin{aligned} \underset{s \nearrow 0}{\text{ lim } } \frac{|\varOmega _s^V \triangle \varOmega |}{s} = -\underset{t \searrow 0}{\text{ lim } } \frac{|\varOmega _{t}^{-V} \triangle \varOmega |}{t} = - \int _{\partial \varOmega } |V \cdot n| \; \text{ d } S_x = -\underset{s \searrow 0}{\text{ lim } } \frac{|\varOmega _{s}^{V} \triangle \varOmega |}{s} \end{aligned}$$

and, assuming differentiability of $s \mapsto \mathfrak g(\varOmega _s^V)$,

$$\begin{aligned} \underset{s \searrow 0}{\text{ lim } } \frac{{\mathfrak {g}}(\varOmega _s^V) - {\mathfrak {g}}(\varOmega )}{|\varOmega _s^V \triangle \varOmega |} = \frac{\underset{s \rightarrow 0}{\text{ lim } } \left( \mathfrak g(\varOmega _s^V) - {\mathfrak {g}}(\varOmega )\right) / s}{\underset{s \searrow 0}{\text{ lim } } |\varOmega _s^V \triangle \varOmega | / s} = -\underset{s \nearrow 0}{\text{ lim } } \frac{{\mathfrak {g}}(\varOmega _s^V) - \mathfrak g(\varOmega )}{|\varOmega _s^V \triangle \varOmega |}. \end{aligned}$$

In the discrete case, however, there may occur situations where the limits in (106) do not coincide. This can be the case in particular in situations where $\phi ({{\textbf{x}}}_k) = 0$. We remark that this issue seemed not to cause problems in our numerical experiments.

**Fig. 7:**
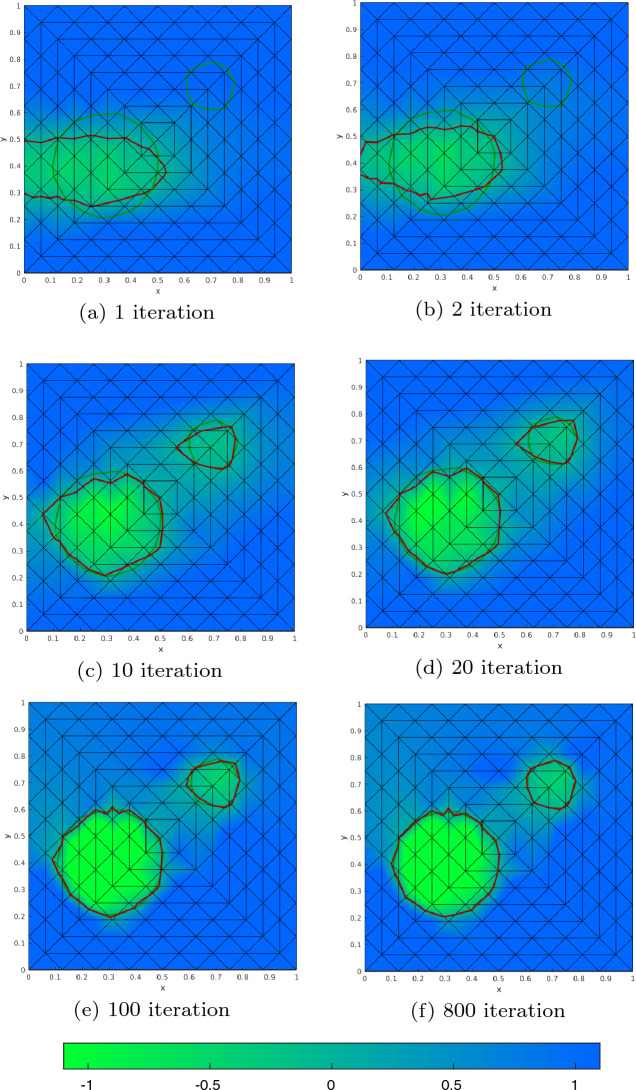
Evolution of the level-set function for the 145 nodes mesh

**Fig. 8:**
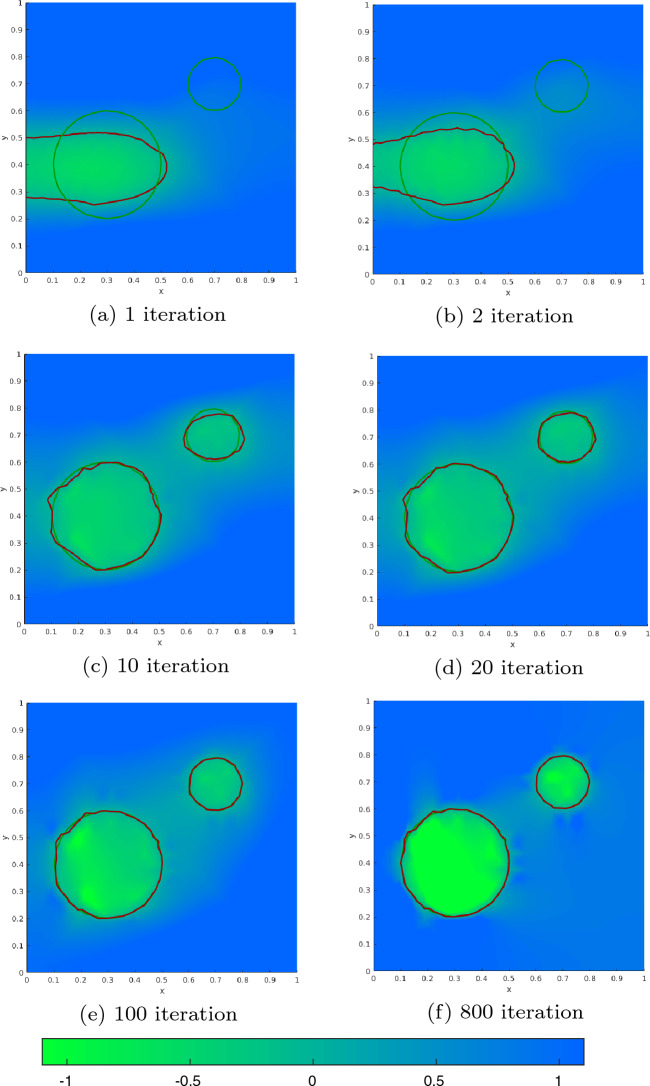
Evolution of the level-set function for the 545 nodes mesh

**Fig. 9:**
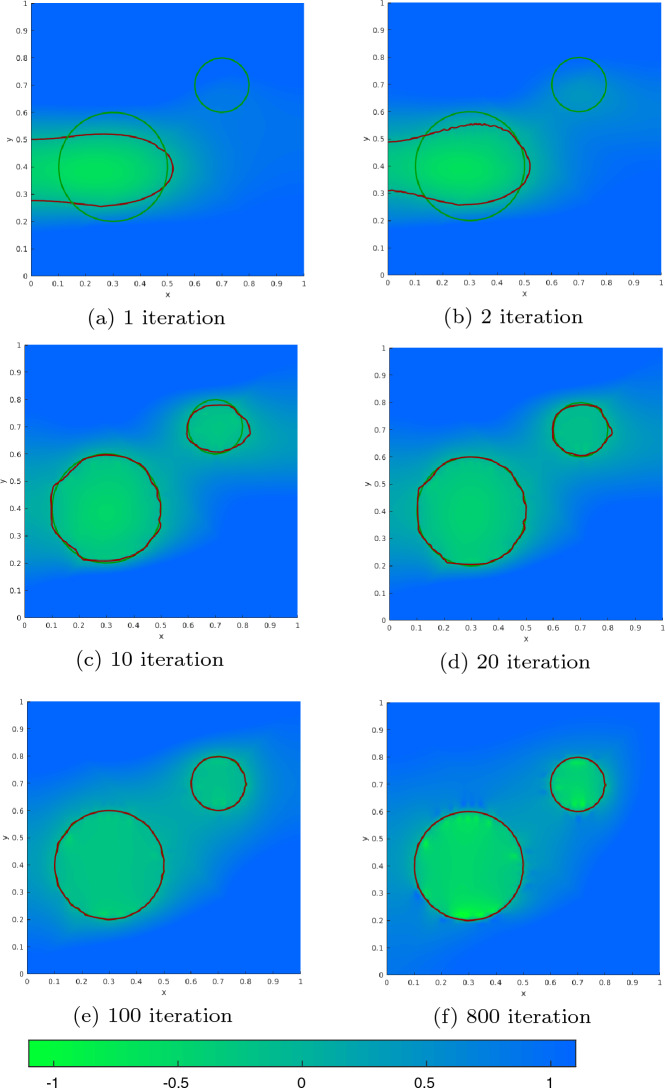
Evolution of the level-set function for the 2113 nodes mesh

**Fig. 10:**
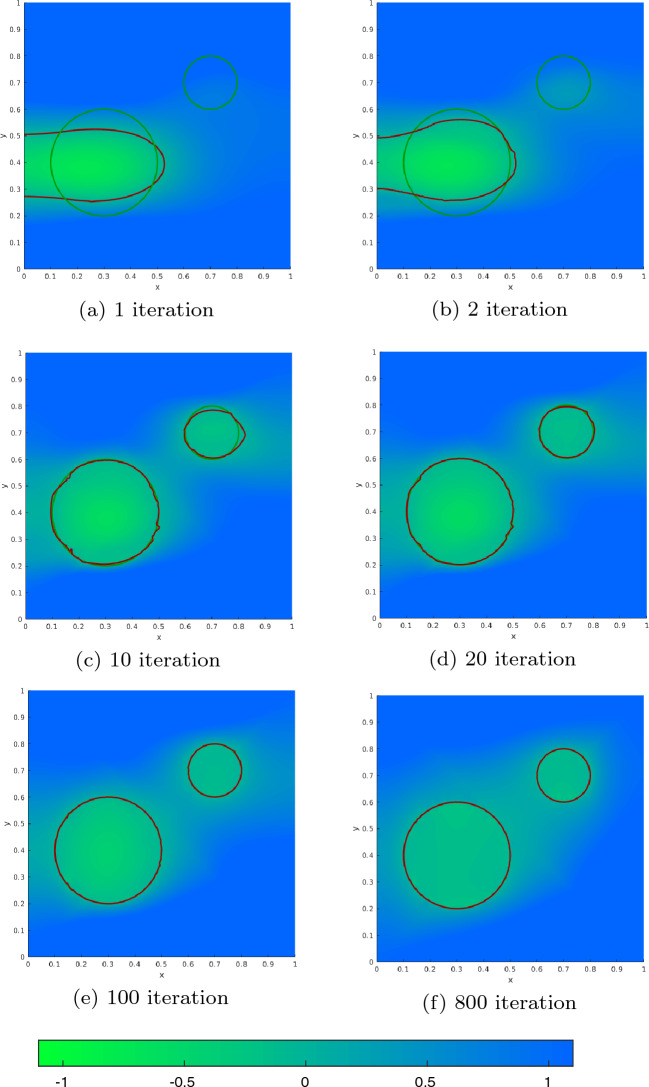
Evolution of the level-set function for the 8321 nodes mesh

**Fig. 11:**
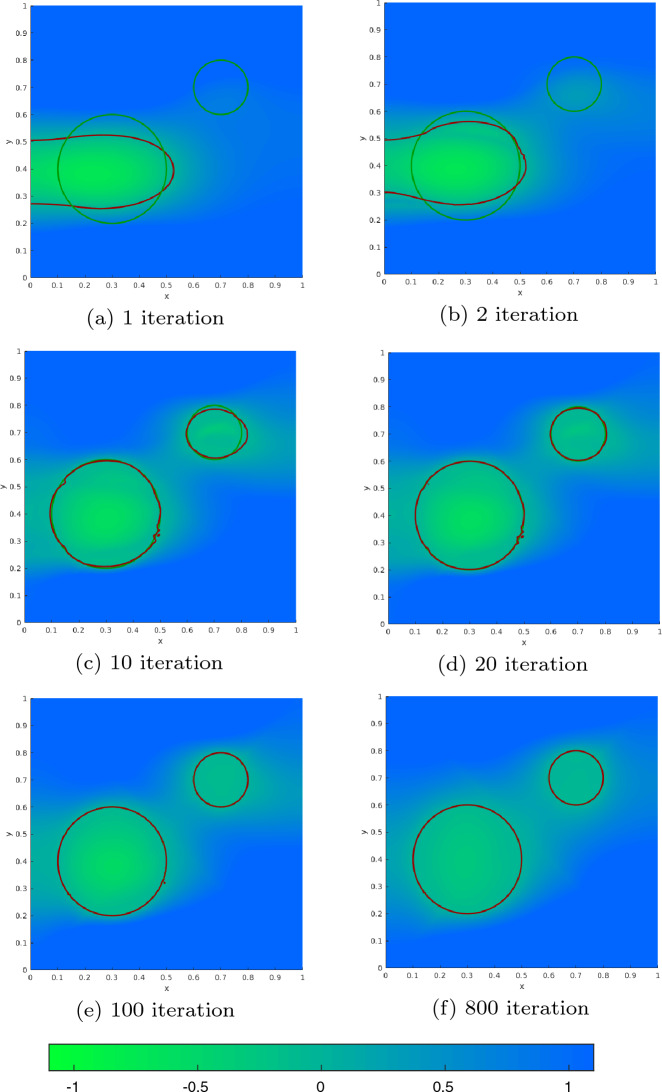
Evolution of the level-set function for the 33025 nodes mesh

**Fig. 12:**
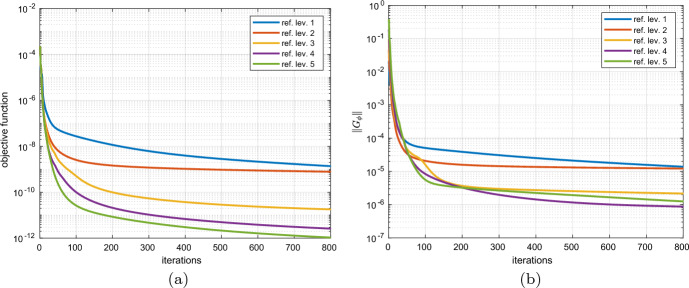
Evolution of the objective function (**a**) and the norm of the topological-shape derivative (**b**) in course of the optimization

##### Remark 12

In practice it turned out to be advantageous to include a smoothing step of the level set function. Thus, we chose the following update strategy: We first set

$$\begin{aligned} \psi = \frac{1}{\sin (\theta _i)}\left( \sin ((1-\kappa _i)\theta _i) \phi _i+ \sin (\kappa _i\theta _i)\frac{G_{\phi _i}}{\Vert G_{\phi _i}\Vert _{L_{2}(D)}} \right) , \end{aligned}$$

with the same notation as above before smoothing the level set function in ${\mathfrak {T}}^-(\psi ) \cup {\mathfrak {T}}^+(\psi )$ by

107 $$\begin{aligned} {\hat{\psi }}({{\textbf{x}}}_k) = {\left\{ \begin{array}{ll} \frac{\sum _{i\in R_{{{\textbf{x}}}_k}} \psi ({{\textbf{x}}}_i) }{|R_{{{\textbf{x}}}_k}|} \quad &{}\text {for } {{\textbf{x}}}_k \in {\mathfrak {T}}^-(\psi )\cup {\mathfrak {T}}^+(\psi ), \\[8pt] \psi ({{\textbf{x}}}_k) \quad &{}\text {for } {{\textbf{x}}}_k \in {\mathfrak {S}}. \end{array}\right. } \end{aligned}$$

Finally, the level-set function is normalized and the next iterate is given by

108 $$\begin{aligned} \phi _{i+1} = \frac{ {\hat{\psi }}}{\Vert {\hat{\psi }}\Vert _{L_{2}(D)}}. \end{aligned}$$

##### Remark 13

Compared to a classical level set method [2] which is based solely on shape sensitivities, our method does not lack a nucleation mechanism and thus it is not necessary to use perforated initial designs or alternating shape or topological update steps [1, 10]. While this is also true for the level set method introduced in [4], which is based solely on topological derivatives, that method typically uses an average of topological derivatives as sensitivities at the material interfaces, which does not necessarily represent the correct shape sensitivities. Moreover, in contrast to these mentioned methods, our approach uses sensitivities of the discretized problem.

#### Numerical Results

As an initial design for the optimisation, we take the empty set, $\varOmega = \emptyset$. This is realized by choosing $\phi _0 = 1/\Vert 1\Vert _{L_2(D)}$ as the initial level set function. We use the algorithm outlined in Sect. 6.2.2 to update this level set function. We terminated the algorithm after the fixed number of 800 iterations. The final as well as some intermediate configurations are illustrated in Figs. 7, 8, 9, 10, and 11 for the five different levels of discretization.

We observe that in all cases the two circles are recovered with high accuracy. In Fig. 12 the evolution of the objective function as well as of the norm of the generalized numerical topological-shape derivative is plotted. We observe that objective function decreases fast and after 800 iterations a reduction by a factor of approximately $10^{-5}-10^{-8}$ could be achieved. Moreover, we observe that the norm of the topological-shape derivative decreases continuously, more and more approaching the optimality condition (102).

## Conclusions

In this work we presented a new sensitivity concept, called the topological-shape derivative which is based on a level set representation of a domain. This approach allows for a unified sensitivity analysis for shape and topological perturbations, which we carried out for a discretized PDE-constrained design optimization problem in two space dimensions. For the discretization we used a standard first order finite element method which does not account for the interface position in the approximation space. Therefore, kinks in the solution of the state and adjoint equations at material interfaces are not resolved. Comparing the computed sensitivities of the discretized problem with the discretization of the continuous topological and shape derivatives, we saw that certain terms did not appear in the former approach. These lack of these terms can be traced back to the inability of the chosen discretization method to represent such kinks. Thus, it would be interesting to study discretization methods which do account for these kinks, *e.g.* XFEM or CutFEM, and perform the sensitivity analysis in these settings in future work. Furthermore, the extension to higher space dimensions, higher polynomial degree and other PDE constraints such as elasticity would be further interesting research directions.

## Figures and Tables

**Fig. 13:**
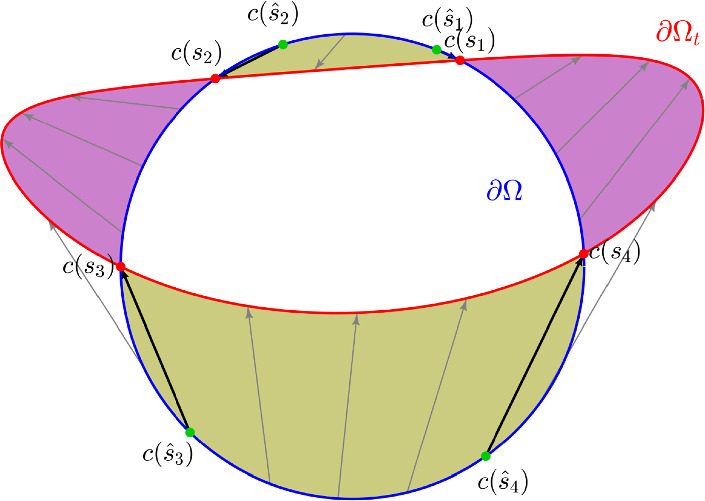
Illustration of the pairs $(s_i(t),\,{\hat{s}}_i(t))$

## Appendix

### Proof of Lemma 1

#### Proof

We investigate the limit

$$\begin{aligned} \underset{t \searrow 0}{\text{ lim }} \frac{1}{t} |\varOmega _t \triangle \varOmega | =&\underset{t \searrow 0}{\text{ lim } } \frac{1}{t} \left( \int _{\varOmega _t\setminus \varOmega }dx + \int _{\varOmega \setminus \varOmega _t} dx\right) . \end{aligned}$$

Let $c:[0,1] \rightarrow \partial \varOmega$ be a smooth parametrization of the boundary $\partial \varOmega$ in counter-clockwise direction with smooth inverse and define $c_t:[0,1] \rightarrow \partial \varOmega _t$,

109 $$\begin{aligned} c_t(s):= c(s) + t V(c(s)) = (\text {id} + t V)(c(s)). \end{aligned}$$

The derivative is given by

110 $$\begin{aligned} {\dot{c_t}}(s) = \frac{d}{ds}c_t(s) = \dot{c}(s) + t \, \partial V(c(s)) \dot{c}(s) = (I + t \, \partial V(c(s))) \dot{c}(s). \end{aligned}$$

Let ${\overline{t}} >0$ sufficiently small such that, for all $t \in (0,{\overline{t}})$, the number of intersection points between $\partial \varOmega$ and $\partial \varOmega _t$ is a fixed number *N*. To each intersection point we associate a pair of numbers $(s_i(t),\,{\hat{s}}_i(t))$ such that

111 $$\begin{aligned} c(s_i(t)) = c_t({\hat{s}}_i(t)) = c({\hat{s}}_i(t)) + t V(c({\hat{s}}_i(t))), \end{aligned}$$

see Fig. 13 for an illustration of the situation. The symmetric difference can now be written as $\varOmega _t \triangle \varOmega = \bigcup _{i=1}^{N} A_i(t)$, where $A_i(t)$ denotes the region between $\partial \varOmega$ and $\partial \varOmega _t$ bounded by the intersection points $c(s_i(t))$ and $c(s_{i+1}(t))$ (here, $A_N(t)$ is bounded by $c(s_N(t))$ and $c(s_1(t))$). More precisely, $A_i(t)$ is bounded by the two segments $\{c(s): s \in [s_{i}(t), s_{i+1}(t)]\}$ and $\{c_t(s): s \in [{\hat{s}}_{i}(t), {\hat{s}}_{i+1}(t)]\}$. Now let $i \in \{1, \dots , N\}$ fixed. The volume of $A_i(t)$ can be expressed by the divergence theorem as

112 $$\begin{aligned}&|A_i(t)| = \int _{A_i(t)} \frac{1}{2}\text{ div } \begin{pmatrix} x_1 \\ x_2 \end{pmatrix} \, \text{ d }x = \frac{1}{2}\int _{\partial A_i(t)} x \cdot n(x) \,\text{ d }S_x \end{aligned}$$

113 $$\begin{aligned}&\quad = \frac{1}{2} \left( \int _{s_i(t)}^{s_{i+1}(t)} c(s)^\top R_i \frac{\dot{c}(s)}{|\dot{c}(s)|} |\dot{c}(s)|\,\text{ d }s + \int _{{\hat{s}}_i(t)}^{{\hat{s}}_{i+1}(t)} c_t( s)^\top (-R_i) \frac{\dot{c}_t( s)}{|{\dot{c_t}}(s)|} |{\dot{c_t}}(s)|\,\text{ d } s \right) \end{aligned}$$

with the rotation matrix

114 $$\begin{aligned} R_i = d_i \begin{bmatrix} 0 &{} 1\\ -1 &{} 0 \end{bmatrix}, \quad \text{ where } d_i = {\left\{ \begin{array}{ll} 1, &{} \text{ if } A_i(t) \subset \varOmega \setminus \varOmega _t, \\ -1, &{}\text{ if } A_i(t) \subset \varOmega _t \setminus \varOmega , \end{array}\right. } \end{aligned}$$

such that $R_i\frac{\dot{c}(s)}{|\dot{c}(s)|}$ and $(-R_i)\frac{\dot{c}_t(s)}{|\dot{c}_t(s)|}$ are the unit normal vectors pointing out of $A_i(t)$ at *c*(*s*) and $c_t(s)$, respectively. Note that $R_i^\top = - R_i$. For further use we note that

$$\begin{aligned} c_t(s)^\top R_i \,{\dot{c_t}}(s)&= [c(s) + t V(c(s))]^\top R_i\,(I + t \, \partial V(c(s)))\,\dot{c}(s) \\&= c(s)^\top R_i \, \dot{c}(s) + t\left( V(c(s))^\top R_i\, \dot{c}(s) \right. \\&\quad \left. + c(s)^\top R_i\cdot \partial V(c(s))\,\dot{c}(s) \right) + t^2 V(s)^\top R_i\,\partial V(c(s))\,\dot{c}(s). \end{aligned}$$

Furthermore, let

115 $$\begin{aligned} {\bar{s}}_i:= \underset{t \searrow 0}{\text{ lim } }s_i(t), \end{aligned}$$

and we observe from (111) that $\underset{t \searrow 0}{\text{ lim } }{\hat{s}}_i(t) = {\bar{s}}_i$.

Moreover, since we assumed the inverse of *c* to be smooth (in particular Lipschitz continuous with constant *L*), we have that the limit

116 $$\begin{aligned} \underset{t \searrow 0}{\text{ lim } }\frac{1}{t} |s_i(t) - {\hat{s}}_i(t)| \le \underset{t \searrow 0}{\text{ lim } }\frac{L}{t} |c(s_i(t)) - c({\hat{s}}_i(t))| = L |V(c({\bar{s}}_i))|, \end{aligned}$$

exists. Here we used (111) and the continuity of *V* and *c*.

With the abbreviation $f_c(s):= c(s)^\top R_i\, \dot{c}(s)$ we have

117 $$\begin{aligned}&|A_i(t)| \nonumber \\&=\frac{1}{2} \Bigg ( \int _{{\hat{s}}_i(t)}^{s_{i}(t)} f_c(s) \,ds + \int _{s_i(t)}^{s_{i+1}(t)} f_c(s) \,ds +\int _{s_{i+1}(t)}^{{\hat{s}}_{i+1}(t)} f_c(s) \,ds- \int _{{\hat{s}}_i(t)}^{{\hat{s}}_{i+1}(t)} f_{c_t}(s) \,d s \nonumber \\&\qquad - \int _{{\hat{s}}_i(t)}^{s_{i}(t)} f_c(s) \,ds - \int _{s_{i+1}(t)}^{{\hat{s}}_{i+1}(t)} f_c(s) \,ds \Bigg ) \nonumber \\&= \frac{1}{2}\Bigg ( \int _{{\hat{s}}_i(t)}^{{\hat{s}}_{i+1}(t)} \big [f_c(s)-f_{c_t}(s) \big ] \,d s - \int _{{\hat{s}}_i(t)}^{s_{i}(t)} f_c(s) \,ds - \int _{s_{i+1}(t)}^{{\hat{s}}_{i+1}(t)} f_c(s) \,ds \Bigg ). \end{aligned}$$

Thus,

$$\frac{1}{t}|A_i(t)| = \frac{1}{2}\big ( B_i(t) - C_i(t) + C_{i+1}(t)\big )$$

with

118 $$\begin{aligned} B_i(t)&=-\int _{{\hat{s}}_i(t)}^{{\hat{s}}_{i+1}(t)} \big [ V(c(s))^\top R_i \, \dot{c}(s) + c(s)^\top R_i \,\partial V(c(s)) \,\dot{c}(s) \big ] \,\text{ d }s + {\mathcal {O}}(t), \end{aligned}$$

119 $$\begin{aligned} C_i(t)&=\frac{1}{t}\int _{{\hat{s}}_i(t)}^{s_i(t)} f_c(s) \, \text{ d }s. \end{aligned}$$

In order to compute $B_i(t)$ we note that

$$\begin{aligned} \frac{d}{ds}(V(c(s))^\top R_i \, c(s))&= V(c(s))^\top R_i \, \dot{c}(s) + (\partial V(c(s)) \dot{c}(s))^\top \, R_i c(s) \\&= V(c(s))^\top R_i \, \dot{c}(s) - c(s)^\top R_i \, \partial V(c(s)) \, \dot{c}(s) \end{aligned}$$

Thus,

120 $$\begin{aligned} B_i(t)&= \int _{{\hat{s}}_i(t)}^{{\hat{s}}_{i+1}(t)} \left[ \frac{d}{ds}(V(c(s))^\top R_i \, c(s)) - 2V(c(s))^\top R_i\, \dot{c}(s) \right] \,ds + {\mathcal {O}}(t) \nonumber \\&= V(c({\hat{s}}_{i+1}(t)))^\top R_i \, c({\hat{s}}_{i+1}(t)) - V(c({\hat{s}}_{i}(t)))^\top R_i \, c({\hat{s}}_{i}(t)) \nonumber \\&\quad - 2 \int _{{\hat{s}}_i(t)}^{{\hat{s}}_{i+1}(t)} V(c(s))^\top R_i \, \dot{c}(s) \, ds+ {\mathcal {O}}(t). \end{aligned}$$

For $C_i(t)$, by the mean value theorem there exists ${\tilde{s}}_i \in [{\hat{s}}_i(t), s_i(t)]$ such that

121 $$\begin{aligned} C_i(t) = \frac{1}{t} \int _{{\hat{s}}_i(t)}^{s_i(t)} c(s)^\top R_i \dot{c}(s) \, \text{ d }s = c({\tilde{s}}_i)^\top R_i \dot{c}({\tilde{s}}_i) \frac{1}{t}|s_i(t) - {\hat{s}}_i(t)|. \end{aligned}$$

On the other hand, we note that with (111) the following vector identity holds

$$\begin{aligned} t V(c({\hat{s}}_{i}(t)))= & {} c(s_{i}(t)) - c({\hat{s}}_{i}(t)) = \int _{{\hat{s}}_{i}(t)}^{s_{i}(t)} \dot{c}(s) \,ds \\= & {} (s_{i}(t)-{\hat{s}}_{i}(t))\int _{0}^{1} \dot{c}({\hat{s}}_{i}(t)+a(s_{i}(t)-{\hat{s}}_{i}(t))) \,da \end{aligned}$$

and thus,

122 $$\begin{aligned}{} & {} \dot{c}({\bar{s}}_{i})\underset{t \searrow 0}{\text{ lim } }\frac{1}{t}(s_{i}(t)-{\hat{s}}_{i}(t)) \nonumber \\{} & {} \quad = \underset{t \searrow 0}{\text{ lim } }\left( \frac{1}{t}(s_{i}(t)-{\hat{s}}_{i}(t))\int _{0}^{1} \dot{c}({\hat{s}}_{i}(t)+a(s_{i}(t)-{\hat{s}}_{i}(t))) \,da \right) = V(c(\bar{s}_{i})).\nonumber \\ \end{aligned}$$

Combining (120) and (121) we get

$$\begin{aligned} \underset{t \searrow 0}{\text{ lim } }\frac{1}{t} |A_i(t)| =&\underset{t \searrow 0}{\text{ lim } }\frac{1}{2} (B_i(t) - C_i(t)+ C_{i+1}(t))\\ =&\underset{t \searrow 0}{\text{ lim } }\frac{1}{2} \left( - 2 \int _{{\hat{s}}_i(t)}^{{\hat{s}}_{i+1}(t)} V(c(s))^\top R_i \, \dot{c}(s) \, ds+ \delta _i(t) - \delta _{i+1}(t) \right) \end{aligned}$$

with

$$\begin{aligned} \delta _i(t)&:= c({\hat{s}}_{i}(t))^\top R_i V(c({\hat{s}}_{i}(t))) - c({\tilde{s}}_i)^\top R_i \dot{c}({\tilde{s}}_i) \frac{1}{t}|s_i(t)- {\hat{s}}_i(t)| . \end{aligned}$$

From (122), it follows that $\underset{t \searrow 0}{\text{ lim } }\delta _i(t) = 0 = \underset{t \searrow 0}{\text{ lim } }\delta _{i+1}(t)$ and thus

$$\begin{aligned} \underset{t \searrow 0}{\text{ lim } }\frac{1}{t} |A_i(t)| =&- \int _{{\bar{s}}_i}^{{\bar{s}}_{i+1}} V(c(s))^\top R_i \, \dot{c}(s) \, ds = - \int _{c({\bar{s}}_i)}^{c(\bar{s}_{i+1})} V(x) \cdot n_i(x) \, \text{ d } S_x \end{aligned}$$

Here we used

$$\begin{aligned} n_i(x) = {\left\{ \begin{array}{ll} n(x) &{} \text{ if } V(x) \cdot n(x) <0, \\ -n(x) &{} \text{ if } V(x) \cdot n(x) >0, \end{array}\right. } \end{aligned}$$

where *n* denotes the unit normal vector pointing out of $\varOmega$. Thus we have found

$$\begin{aligned} \underset{t \searrow 0}{\text{ lim } }\frac{1}{t} |\varOmega _t \triangle \varOmega | = \sum _{i=1}^N \underset{t \searrow 0}{\text{ lim } }\frac{1}{t} |A_i(t)| = \int _{\partial \varOmega } |V(x) \cdot n(x)| \; \text{ d }S_x. \end{aligned}$$

$$\square $$

### Matrix Entries for the Numerical Shape Derivative

In order to simplify the notation we use in this section the abbreviation $\phi _{i}:=\phi _{l_i}$ for $i=1,2,3$. The matrix entries for $d_k{\textbf{m}}_{l}^I = \left| {\text {det} }J_l\right| d_k\bar{\textbf{m}}_{l}^I$ and $d_k{\textbf{f}}_{l}^I = \left| {\text {det} }J_l\right| d_k\bar{{\textbf{f}}}_{l}^I$ in (66) are given by

$$\begin{aligned}&d_k{\bar{m}}_l^{A^\pm }[1,1]\\&\quad =\pm \frac{\phi _{1}^4\left( \phi _{2}^3+\phi _{2}^2\phi _{3} +\phi _{2}\phi _{3}^2+\phi _{3}^3\right) -4\phi _{1}\phi _{2}^3\phi _{3}^3 +6\phi _{1}^2\phi _{2}^2\phi _{3}^2\left( \phi _{2}+\phi _{3}\right) -4\phi _{1}^3\phi _{2}\phi _{3} \left( \phi _{2}^2+\phi _{2}\phi _{3}+\phi _{3}^2\right) }{4{\left( \phi _{1}-\phi _{2}\right) }^4 {\left( \phi _{1}-\phi _{3}\right) }^4}, \end{aligned}$$

$$\begin{aligned} d_k{\bar{m}}_l^{A^\pm }[1,2]&= d_k{\bar{m}}_l^{A^\pm }[2,1] \\&= \mp \frac{\phi _{1}^2\left( 3\phi _{1}^2\phi _{2}^2+2\phi _{1}^2\phi _{2}\phi _{3}+\phi _{1}^2\phi _{3}^2-8\phi _{1}\phi _{2}^2\phi _{3}-4\phi _{1}\phi _{2}\phi _{3}^2+6\phi _{2}^2\phi _{3}^2\right) }{12{\left( \phi _{1}-\phi _{2}\right) }^4{\left( \phi _{1}-\phi _{3}\right) }^3},\\ d_k{\bar{m}}_l^{A^\pm }[1,3]&= d_k{\bar{m}}_l^{A^\pm }[3,1]\\&= \mp \frac{\phi _{1}^2\left( \phi _{1}^2\phi _{2}^2+2\phi _{1}^2\phi _{2}\phi _{3}+3\phi _{1}^2\phi _{3}^2-4\phi _{1}\phi _{2}^2\phi _{3}-8\phi _{1}\phi _{2}\phi _{3}^2+6\phi _{2}^2\phi _{3}^2\right) }{12{\left( \phi _{1}-\phi _{2}\right) }^3{\left( \phi _{1}-\phi _{3}\right) }^4},\\ d_k{\bar{m}}_l^{A^\pm }[2,2]&=\pm \frac{\phi _{1}^3\left( 3\phi _{1}\phi _{2}+\phi _{1}\phi _{3}-4\phi _{2}\phi _{3}\right) }{12{\left( \phi _{1}-\phi _{2}\right) }^4{\left( \phi _{1}-\phi _{3}\right) }^2},\\ d_k{\bar{m}}_l^{A^\pm }[2,3]&= d_k{\bar{m}}_l^{A^\pm }[3,2]= \pm \frac{\phi _{1}^3\left( \phi _{1}\phi _{2}+\phi _{1}\phi _{3}-2\phi _{2}\phi _{3}\right) }{12{\left( \phi _{1}-\phi _{2}\right) }^3{\left( \phi _{1}-\phi _{3}\right) }^3},\\ d_k{\bar{m}}_l^{A^\pm }[3,3]&= \pm \frac{\phi _{1}^3\left( \phi _{1}\phi _{2}+3\phi _{1}\phi _{3}-4\phi _{2}\phi _{3}\right) }{12{\left( \phi _{1}-\phi _{2}\right) }^2{\left( \phi _{1}-\phi _{3}\right) }^4}, \\ d_k{\bar{m}}_l^{B^\pm }[1,1]&=\mp \frac{\phi _{2}^4}{4{\left( \phi _{1}-\phi _{2}\right) }^4\left( \phi _{2}-\phi _{3}\right) },\\ d_k{\bar{m}}_l^{B^\pm }[1,2]&= d_k{\bar{m}}_l^{B^\pm }[2,1]= \pm \frac{\phi _{2}^3\left( 3\phi _{1}\phi _{2}-4\phi _{1}\phi _{3}+\phi _{2}\phi _{3}\right) }{12{\left( \phi _{1}-\phi _{2}\right) }^4{\left( \phi _{2}-\phi _{3}\right) }^2},\\ d_k{\bar{m}}_l^{B^\pm }[1,3]&= d_k{\bar{m}}_l^{B^\pm }[3,1]= \pm \frac{\phi _{2}^4}{12{\left( \phi _{1}-\phi _{2}\right) }^3{\left( \phi _{2}-\phi _{3}\right) }^2},\\ d_k{\bar{m}}_l^{B^\pm }[2,2]&=\mp \frac{\phi _{2}^2\left( 3\phi _{1}^2\phi _{2}^2-8\phi _{1}^2\phi _{2}\phi _{3}+6\phi _{1}^2\phi _{3}^2+2\phi _{1}\phi _{2}^2\phi _{3}-4\phi _{1}\phi _{2}\phi _{3}^2+\phi _{2}^2\phi _{3}^2\right) }{12{\left( \phi _{1}-\phi _{2}\right) }^4{\left( \phi _{2}-\phi _{3}\right) }^3},\\ d_k{\bar{m}}_l^{B^\pm }[2,3]&= d_k{\bar{m}}_l^{B^\pm }[3,2]= \mp \frac{\phi _{2}^3\left( \phi _{1}\phi _{2}-2\phi _{1}\phi _{3}+\phi _{2}\phi _{3}\right) }{12{\left( \phi _{1}-\phi _{2}\right) }^3{\left( \phi _{2}-\phi _{3}\right) }^3},\\ d_k{\bar{m}}_l^{B^\pm }[3,3]&=\mp \frac{\phi _{2}^4}{12{\left( \phi _{1}-\phi _{2}\right) }^2{\left( \phi _{2}-\phi _{3}\right) }^3}, \\ d_k{\bar{f}}_l^{A^\pm }[1]&=\mp \frac{\phi _{1}\left( \phi _{1}^2\phi _{2}^2+\phi _{1}^2\phi _{2}\phi _{3}+\phi _{1}^2\phi _{3}^2-3\phi _{1}\phi _{2}^2\phi _{3}-3\phi _{1}\phi _{2}\phi _{3}^2+3\phi _{2}^2\phi _{3}^2\right) }{3{\left( \phi _{1}-\phi _{2}\right) }^3{\left( \phi _{1}-\phi _{3}\right) }^3},\\ d_k\bar{f}_l^{A^\pm }[2]&=\pm \frac{\phi _{1}^2\left( 2\phi _{1}\phi _{2}+\phi _{1}\phi _{3}-3\phi _{2}\phi _{3}\right) }{6{\left( \phi _{1}-\phi _{2}\right) }^3{\left( \phi _{1}-\phi _{3}\right) }^2},\\ d_k{\bar{f}}_l^{A^\pm }[3]&=\pm \frac{\phi _{1}^2\left( \phi _{1}\phi _{2}+2\phi _{1}\phi _{3}-3\phi _{2}\phi _{3}\right) }{6{\left( \phi _{1}-\phi _{2}\right) }^2{\left( \phi _{1}-\phi _{3}\right) }^3}, \\ d_k\bar{f}_l^{B^\pm }[1]&=\pm \frac{\phi _{2}^3}{3{\left( \phi _{1}-\phi _{2}\right) }^3\left( \phi _{2}-\phi _{3}\right) },\\ d_k{\bar{f}}_l^{B^\pm }[2]&=\mp \frac{\phi _{2}^2\left( 2\phi _{1}\phi _{2}-3\phi _{1}\phi _{3}+\phi _{2}\phi _{3}\right) }{6{\left( \phi _{1}-\phi _{2}\right) }^3{\left( \phi _{2}-\phi _{3}\right) }^2}, \\ d_k\bar{f}_l^{B^\pm }[3]&=\mp \frac{\phi _{2}^3}{6{\left( \phi _{1}-\phi _{2}\right) }^2{\left( \phi _{2}-\phi _{3}\right) }^2}. \end{aligned}$$

The formulas for $d_k{\bar{m}}_l^{C^\pm }$ and $d_k\bar{f}_l^{C^\pm }$ can be obtained from the formulas for $d_k\bar{m}_l^{B^\pm }$ and $d_k{\bar{f}}_l^{B^\pm }$ by exchanging the matrix entry indices 2 and 3, and $\phi _{2}$ and $\phi _{3}$, *e.g.* $d_k\bar{f}_l^{C^\pm }[3]$ is obtained from $d_k{\bar{f}}_l^{B^\pm }[2]$ by exchanging $\phi _{2}$ and $\phi _{3}$.

## References

[1] Allaire G, Jouve F, Bendsøe MP, Olhoff N, Sigmund O (2006). Coupling the level set method and the topological gradient in structural optimization. IUTAM Symposium on Topological Design Optimization of Structures, Machines and Materials.

[2] Allaire G, Jouve F, Toader AM (2004). Structural optimization using sensitivity analysis and a level-set method. J. Comput. Phys..

[3] Amstutz, S.: Sensitivity analysis with respect to a local perturbation of the material property. Asymptot. Anal. **49**(1,2), 87–108 (2006)

[4] Amstutz S, Andrä H (2006). A new algorithm for topology optimization using a level-set method. J. Comput. Phys..

[5] Amstutz S, Gangl P (2019). Topological derivative for the nonlinear magnetostatic problem. Electron. Trans. Numer. Anal..

[6] Amstutz S, Dapogny C, Ferrer À (2018). A consistent relaxation of optimal design problems for coupling shape and topological derivatives. Numer. Math..

[7] Bendsoe M, Sigmund O (2003). Topology Optimization: Theory, Methods, and Applications.

[8] Berggren, M.: Shape calculus for fitted and unfitted discretizations: domain transformations vs. boundary-face dilations. (2022). 10.48550/ARXIV.2210.10411

[9] Bernland A, Wadbro E, Berggren M (2018). Acoustic shape optimization using cut finite elements. Int. J. Numer. Methods Eng..

[10] Burger M, Hackl B, Ring W (2004). Incorporating topological derivatives into level set methods. J. Comput. Phys..

[11] Burman E, Claus S, Hansbo P, Larson MG, Massing A (2015). Cutfem: discretizing geometry and partial differential equations. Int. J. Numer. Meth. Eng..

[12] Dapogny, C., Feppon, F.: Shape optimization using a level set based mesh evolution method: an overview and tutorial (2022). https://hal.science/hal-03881641

[13] Delfour MC (2018). Topological derivative: a semidifferential via the Minkowski content. J. Convex Anal..

[14] Delfour MC (2022). Topological derivatives via one-sided derivative of parametrized minima and minimax. Eng. Comput..

[15] Delfour, M.C., Zolésio, J.P.: Shapes and geometries: metrics, analysis, differential calculus, and optimization. In: Advances in Design and Control, vol. 22, 2nd edn. Society for Industrial and Applied Mathematics (SIAM), Philadelphia, PA (2011)

[16] Fike, J., Alonso, J.: The development of hyper-dual numbers for exact second-derivative calculations. In: 49th AIAA Aerospace Sciences Meeting including the New Horizons Forum and Aerospace Exposition, p. 886 (2011)

[17] Gangl P (2020). A multi-material topology optimization algorithm based on the topological derivative. Comput. Methods Appl. Mech. Eng..

[18] Gangl, P., Sturm, K.: A simplified derivation technique of topological derivatives for quasi-linear transmission problems. ESAIM **26**, 106 (2020)

[19] Gangl P, Langer U, Laurain A, Meftahi H, Sturm K (2015). Shape optimization of an electric motor subject to nonlinear magnetostatics. SIAM J. Sci. Comput..

[20] Hägg L, Wadbro E (2018). On minimum length scale control in density based topology optimization. Struct. Multidiscip. Optim..

[21] Haubner J, Siebenborn M, Ulbrich M (2021). A continuous perspective on shape optimization via domain transformations. SIAM J. Sci. Comput..

[22] Laurain A (2018). Analyzing smooth and singular domain perturbations in level set methods. SIAM J. Math. Anal..

[23] Laurain, A., Sturm, K.: Distributed shape derivative via averaged adjoint method and applications. ESAIM **50**(4), 1241–1267 (2016)

[24] Martins JR, Sturdza P, Alonso JJ (2003). The complex-step derivative approximation. ACM Trans. Math. Softw..

[25] Moës N, Dolbow J, Belytschko T (1999). A finite element method for crack growth without remeshing. Int. J. Numer. Meth. Eng..

[26] Novotny A, Sokolowski J (2013). Topological Derivatives in Shape Optimization. Interaction of Mechanics and Mathematics.

[27] Novotny A, Feijóo R, Taroco E, Padra C (2003). Topological sensitivity analysis. Comput. Methods Appl. Mech. Eng..

[28] Novotny A, Feijóo R, Taroco E, Padra C, Bendsøe MP, Olhoff N, Sigmund O (2006). Topological-shape sensitivity method: theory and applications. IUTAM Symposium on Topological Design Optimization of Structures, Machines and Materials.

[29] Sigmund O, Maute K (2013). Topology optimization approaches. Struct. Multidiscip. Optim..

[30] Sokolowski J, Zochowski A (1999). On the topological derivative in shape optimization. SIAM J. Control. Optim..

[31] Sturm K (2015). Minimax Lagrangian approach to the differentiability of nonlinear PDE constrained shape functions without saddle point assumption. SIAM J. Control. Optim..

